# Effectiveness of high cardiorespiratory fitness in cardiometabolic protection in prediabetic rats

**DOI:** 10.1186/s10020-022-00458-9

**Published:** 2022-03-10

**Authors:** Chanisa Thonusin, Patcharapong Pantiya, Natticha Sumneang, Titikorn Chunchai, Wichwara Nawara, Busarin Arunsak, Natthaphat Siri-Angkul, Sirawit Sriwichaiin, Siriporn C. Chattipakorn, Nipon Chattipakorn

**Affiliations:** 1 Cardiac Electrophysiology Unit, Department of Physiology, Faculty of Medicine, Chiang Mai University, Chiang Mai, Thailand; 2 Cardiac Electrophysiology Research and Training Center, Faculty of Medicine, Chiang Mai University, Chiang Mai, Thailand; 3 Center of Excellence in Cardiac Electrophysiology Research, Chiang Mai University, Chiang Mai, Thailand; 4 Department of Oral Biology and Diagnostic Sciences, Faculty of Dentistry, Chiang Mai University, Chiang Mai, Thailand

**Keywords:** Prediabetes, Lifestyle modification, Weight maintenance, Cardiorespiratory fitness, Cardiometabolic protection

## Abstract

**Background:**

Caloric restriction and exercise are lifestyle interventions that effectively attenuate cardiometabolic impairment. However, cardioprotective effects of long-term lifestyle interventions and short-term lifestyle interventions followed by weight maintenance in prediabetes have never been compared. High cardiorespiratory fitness (CRF) has been shown to provide protection against prediabetes and cardiovascular diseases, however, the interactions between CRF, prediabetes, caloric restriction, and exercise on cardiometabolic health has never been investigated.

**Methods:**

Seven-week-old male Wistar rats were fed with either a normal diet (ND; n = 6) or a high-fat diet (HFD; n = 30) to induce prediabetes for 12 weeks. Baseline CRF and cardiometabolic parameters were determined at this timepoint. The ND-fed rats were fed continuously with a ND for 16 more weeks. The HFD-fed rats were divided into 5 groups (n = 6/group) to receive one of the following: (1) a HFD without any intervention for 16 weeks, (2) 40% caloric restriction for 6 weeks followed by an ad libitum ND for 10 weeks, (3) 40% caloric restriction for 16 weeks, (4) a HFD plus an exercise training program for 6 weeks followed by a ND without exercise for 10 weeks, or (5) a HFD plus an exercise training program for 16 weeks. At the end of the interventions, CRF and cardiometabolic parameters were re-assessed. Then, all rats were euthanized and heart tissues were collected.

**Results:**

Either short-term caloric restriction or exercise followed by weight maintenance ameliorated cardiometabolic impairment in prediabetes, as indicated by increased insulin sensitivity, improved blood lipid profile, improved mitochondrial function and oxidative phosphorylation, reduced oxidative stress and inflammation, and improved cardiac function. However, these benefits were not as effective as those of either long-term caloric restriction or exercise. Interestingly, high-level baseline CRF was correlated with favorable cardiac and metabolic profiles at follow-up in prediabetic rats, both with and without lifestyle interventions.

**Conclusions:**

Short-term lifestyle modification followed by weight maintenance improves cardiometabolic health in prediabetes. High CRF exerted protection against cardiometabolic impairment in prediabetes, both with and without lifestyle modification. These findings suggest that targeting the enhancement of CRF may contribute to the more effective treatment of prediabetes-induced cardiometabolic impairment.

**Supplementary Information:**

The online version contains supplementary material available at 10.1186/s10020-022-00458-9.

## Background

Prediabetes is defined as an intermediate state of hyperglycemia, in which the glycemic parameters are higher than normal but lower than the diabetic criteria (Bansal 2015). This condition is strongly associated with hyperinsulinemia, as a result of insulin resistance (Brannick and Dagogo-Jack 2018). Moreover, prediabetes commonly develops in conjunction with a variety of metabolic abnormalities including obesity, hypertension, and dyslipidemia (Mahat et al. 2019). Together this cluster of conditions is termed ‘metabolic syndrome’ (Rochlani et al. 2017). Regarding the prevalence of prediabetes, the International Diabetes Federation predicts a global increase in its prevalence to 471 million people by 2035 (Bansal 2015). Additionally, it has been shown that approximately 5–10% of people with prediabetes become type 2 diabetic every year (Forouhi et al. 2007). Importantly, a recent meta-analysis revealed that prediabetes was associated with increased risk of all-cause mortality and cardiovascular diseases, both in the general population and in patients with atherosclerotic cardiovascular disease (Cai et al. 2020). For this reason, not only type 2 diabetes, but prediabetes should also be recognized as a global public health concern.

It has been shown that lifestyle modification, including caloric restriction and exercise, are safe, non-invasive interventions that effectively attenuate prediabetes via an improvement of insulin sensitivity (Hill 2006; Khaodhiar et al. 2009). These interventions can also delay the development of type 2 diabetes (Khaodhiar et al. 2009) and decrease the risks of cardiovascular diseases (Brannick and Dagogo-Jack 2018). Unfortunately, it is difficult to maintain caloric restriction and exercise for a whole life span due to physiological adaptations for starvation (Collins 1995) and diminished exercise performance with age (Ganse et al. 2018), respectively. Due to these adaptations, approximately 30%–35% of lost weight is regained after one year, and 50% of people return to their initial weight within five years after successful weight loss (Sarwer et al. 2009). Since long-term lifestyle modification is not practical in many people, short-term lifestyle modification followed by weight maintenance seems to be the most feasible non-medical interventions to ameliorate prediabetes and to reduce the risk of cardiovascular disease. However, comparisons of the cardioprotective effects between long-term lifestyle interventions and short-term lifestyle interventions followed by weight maintenance in prediabetes have never been investigated.

Since life-long caloric restriction and exercise are rarely feasible, an alternative strategy that exerts protection against prediabetes and cardiovascular disease is a topic of interest to us. Such a strategy is ‘cardiorespiratory fitness’. Cardiorespiratory fitness (CRF) is defined as the ability of the circulatory, respiratory, and muscular systems to supply sufficient oxygen during sustained physical activity (D. C. Lee et al. 2010). Interestingly, a previous meta-analysis showed that CRF is highly genetically determined, in which it exhibited a weighted heritability estimate of 72% (Schutte et al. 2016). A number of previous longitudinal studies demonstrated that high CRF resulted in a decreased incidence of metabolic syndrome (Brien et al. 2007; Carnethon et al. 2003) as well as a reduction in a variety of cardiovascular diseases, including ischemic heart disease, heart failure, and cardiac arrhythmia (Artero et al. 2014; Kodama et al. 2009; Qureshi et al. 2015). Nevertheless, the beneficial effects of high CRF have never been compared between a normal population, prediabetic patients, and prediabetic individuals who currently receive lifestyle modifications. When considering the molecular mechanisms which mediate the beneficial effects of high CRF on the heart, prior studies in rats selectively bred for high and low intrinsic running capacity reported that high CRF was protective against cardiovascular diseases via the modulation of cardiac mitochondrial respiration, redox reaction capacity, cardiomyocyte contractility, cardiac fibrosis, cardiac remodeling, cardiac systolic function, and cardiac diastolic function (Hussain et al. 2001; Koch et al. 2011; Ritchie et al. 2013; Souza et al. 2018). However, the molecular modulation of CRF on the heart have neither been identified in non-selectively bred rats for intrinsic running capacity nor been compared between the normal condition, prediabetes, and prediabetes with lifestyle modification. In other words, the interaction between CRF, prediabetes, caloric restriction, and exercise on the heart has never been investigated.

This study aimed to compare the beneficial effects of long-term lifestyle modification and short-term lifestyle modification followed by weight maintenance on the attenuation of cardiometabolic impairment in prediabetic rats. In addition, we aimed to compare the effects of high CRF on protection against cardiometabolic impairment in normal rats, prediabetic rats, and prediabetic rats receiving lifestyle modifications. Our findings will contribute to the establishment of new clinical practice guidelines regarding lifestyle modifications for prediabetic patients. We also anticipate that our results regarding the benefits of high CRF for cardioprotection against prediabetes will lead to the development of novel therapeutic paradigms that have direct impact on increasing intrinsic CRF level.

## Methods

### Animals

All experiments related to animals were ethically approved by the laboratory animal center, Chiang Mai University, Chiang Mai, Thailand, and were carried out in accordance with the guidelines established by US National Research Council 2010 (Guide for the care and use of laboratory animals). Male Wistar rats (n = 36) were bought from the Nomura Siam International Co. ltd. (Bangkok, Thailand). All rats were individually housed in a temperature-controlled environment with a 12:12 h light:dark cycle. Food intake and body weight were recorded daily and weekly, respectively.

### Study protocol

**Fig. 1:**
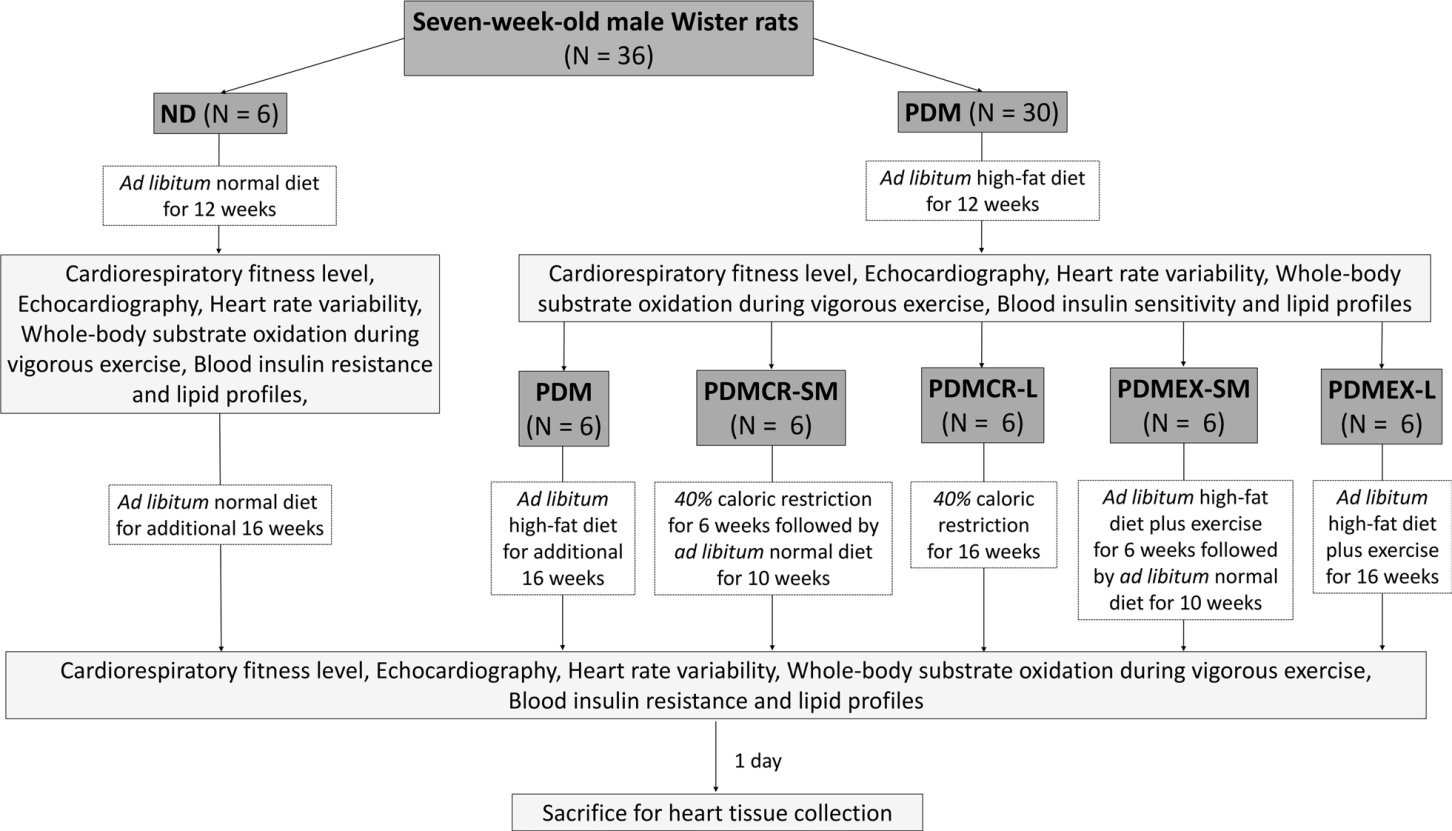
The experimental protocol. ND: Normal diet; PDM: Prediabetes with no intervention; PDMCR-SM: Prediabetes with short-term caloric restriction followed by weight maintenance; PDMCR-L: Prediabetes with long-term caloric restriction; PDMEX-SM: Prediabetes with short-term exercise followed by weight maintenance; PDMEX-L: Prediabetes with long-term exercise

The study protocol is illustrated in Fig. 1. Seven-week-old male Wistar rats (n = 36) were initially divided into 2 groups. The first group (n = 6) received a normal diet (ND) ad libitum containing 19.7% energy from fat with the total energy of 4.02 kCal/g for 12 weeks (ND group). The second group (n = 30) received a high-fat diet (HFD) ad libitum containing 59.28% energy from fat with the total energy of 5.35 kCal/g for 12 weeks to induce prediabetes (PDM group). The compositions of ND and HFD have been previously described elsewhere (Pratchayasakul et al. 2011; Thonusin et al. 2019). At the end of week 12, CRF level was measured as a baseline value. To identify cardiometabolic status at baseline, echocardiography, heart rate variability (HRV), whole-body substrate oxidation during vigorous exercise, blood insulin resistance profile, and blood lipid profile were also investigated at this timepoint.

At the beginning of week 13, all rats from the ND group were continuously fed with a ND ad libitum for an additional 16 weeks. Meanwhile, the rats from the PDM group were equally divided into 5 groups (n = 6 per group). The first group received an ad libitum HFD for an additional 16 weeks (PDM group). The second group received 40% caloric restriction in the form of ND for 6 weeks, followed by an ad libitum ND for an additional 10 weeks as a weight maintenance phase (PDMCR-SM group). The third group received 40% caloric restriction in the form of ND for an additional 16 weeks (PDMCR-L group). The fourth group was fed continuously with an ad libitum HFD plus an exercise training program for 30 min/day at a frequency of 5 days/week for 6 weeks, followed by an ad libitum ND without exercise for an additional 10 weeks as a weight maintenance phase (PDMEX-SM group). The last group was continuously fed with an ad libitum HFD plus an exercise training program for 30 min/day at a frequency of 5 days/week for an additional 16 weeks (PDMEX-L group). At the end of the 16-week interventions (week 28 of the experiment), the CRF level and cardiometabolic parameters were re-evaluated. After that, all rats were euthanized after 12 h of fasting and heart tissue samples were collected. Human regular insulin (Actrapid® HM, Novo Nordisk, Bagsværd, Copenhagen, Denmark) at a dose of 10 U/kg was intraperitoneally injected into each rat at 30 min before euthanasia to stimulate the cardiac insulin signaling pathway.

### Cardiorespiratory fitness (CRF) measurement

CRF level was measured as a running distance value using a speed-ramped treadmill exercise test to exhaustion protocol as described previously (Biesiadecki et al. 2020). Briefly, a five-lane rodent treadmill with electric shock (Panlab/Harvad Instruments, Barcelona, Spain) was set at an inclination of 15˚ with an initial speed of 16 cm/sec and 2 mA of electrical stimulation. The speed was increased automatically at the rate of 2 cm/sec every 2 min. The experiment was stopped when the rat developed exhaustion, as indicated by at least 3 instances of a 2-s drop-down period. Each rat ran on the treadmill for five consecutive days. CRF level was reported as the best running distance out of the five days of the test.

### Whole-body substrate oxidation during vigorous exercise

Whole-body substrate oxidation, including carbohydrate oxidation and fatty acid oxidation were determined by an indirect calorimetric method during a speed-ramped treadmill exercise test to exhaustion. A closed-system treadmill with gas analyzer (the OxyletPro, Panlab/Harvad Instruments, Barcelona, Spain) was set at an inclination of 15˚ with an initial speed of 16 cm/sec and 2-mA electrical stimulation. The speed was automatically increased at the rate of 2 cm/sec every 2 min. During the experiment, the oxygen uptake (VO_2_) and the volume of exhaled carbon dioxide (VCO_2_) were continuously recorded using Metabolism software (Panlab/Harvad Instruments, Barcelona, Spain). The experiment was stopped when the rat developed exhaustion, as indicated by at least 3 instances of a 2-s drop-down period. Carbohydrate and fatty acid oxidation rates during vigorous exercise were calculated using VO_2_ and VCO_2_ at 75% of VO_2_max (Gormley et al. 2008; Overmyer et al. 2015) using the following equations: (Farinatti et al. 2016; Gonzalez et al. 2014)

$${\text{Carbohydrate}\,{\text{oxidation} }}\left( {{{{\text{mg}}} \mathord{\left/ {\vphantom {{{\text{mg}}} {{{{\text{kg}}} \mathord{\left/ {\vphantom {{{\text{kg}}} {{\text{min}}}}} \right. \kern-\nulldelimiterspace} {{\text{min}}}}}}} \right. \kern-\nulldelimiterspace} {{{{\text{kg}}} \mathord{\left/ {\vphantom {{{\text{kg}}} {{\text{min}}}}} \right. \kern-\nulldelimiterspace} {{\text{min}}}}}}} \right) = \frac{{4.120 \times {\text{VCO}}_{2} \left( {{{{\text{mL}}} \mathord{\left/ {\vphantom {{{\text{mL}}} {{\text{min}}}}} \right. \kern-\nulldelimiterspace} {{\text{min}}}}} \right){-}2.962 \times {\text{VO}}_{2} \left( {{{{\text{mL}}} \mathord{\left/ {\vphantom {{{\text{mL}}} {{\text{min}}}}} \right. \kern-\nulldelimiterspace} {{\text{min}}}}} \right)}}{{{\text{bodyweight }}\,({\text{kg}})}}$$

$${\text{Fatty acid oxidation}}\left( {{{{\text{mg}}} \mathord{\left/ {\vphantom {{{\text{mg}}} {{{{\text{kg}}} \mathord{\left/ {\vphantom {{{\text{kg}}} {{\text{min}}}}} \right. \kern-\nulldelimiterspace} {{\text{min}}}}}}} \right. \kern-\nulldelimiterspace} {{{{\text{kg}}} \mathord{\left/ {\vphantom {{{\text{kg}}} {{\text{min}}}}} \right. \kern-\nulldelimiterspace} {{\text{min}}}}}}} \right) = \frac{{1.695 \times {\text{VO}}_{2} \left( {{{{\text{mL}}} \mathord{\left/ {\vphantom {{{\text{mL}}} {{\text{min}}}}} \right. \kern-\nulldelimiterspace} {{\text{min}}}}} \right) - 1.071 \times {\text{VCO}}_{2} \left( {{{{\text{mL}}} \mathord{\left/ {\vphantom {{{\text{mL}}} {{\text{min}}}}} \right. \kern-\nulldelimiterspace} {{\text{min}}}}} \right)}}{{{\text{bodyweight }}({\text{kg}})}}$$

#### Caloric restriction protocol

Caloric restriction protocol was set based upon our previous reports, in which 40% caloric restriction led to a significant improvement of cardiometabolic profile in prediabetic rats (Palee et al. 2019; Tanajak et al. 2017). Briefly, the caloric intake (kCal/day) during 12 weeks of HFD feeding was calculated for each rat. During the caloric restriction period, each rat received 60% of its previous daily caloric intake in the form of normal diet.

#### Exercise training protocol

Exercise training was performed on a 5-lane rodent treadmill with electric shock (Panlab/Harvad Instruments, Barcelona, Spain). The treadmill was set at 0˚ inclination with an initial speed of 8 cm/sec and a step up of 2 cm/sec per day each day until it reached 20 cm/sec. The duration of exercise was 30 min/day with a frequency of 5 days/week (J. Lee et al. 2014; Wang et al. 2020).

#### Plasma insulin resistance profile

Fasting blood was collected from the tail vein after 5-h fasting. Fasting plasma glucose was determined using a colorimetric assay kit (ERBA Mannheim, Mannheim, Germany). Insulin level was measured using a sandwich enzyme-linked immunosorbent assay (ELISA) kit (LINCO Research, Saint Charles, MO, USA). To identify the degree of insulin resistance, the homeostatic model assessment of insulin resistance (HOMA-IR) was calculated using the following equation: (Yokoyama et al. 2004)

$$\mathrm{HOMA}-\mathrm{IR }= \frac{\mathrm{Fasting \,glucose }\,(\mathrm{mg}/\mathrm{dL}) \times \mathrm{Fasting \, insulin }\,(\mathrm{mU}/\mathrm{mL}) }{405}$$

#### Plasma lipid profile

$$\mathrm{Calculated \,LDL }\,\left(\mathrm{mg}/\mathrm{dL}\right)=\mathrm{ Total \,cholesterol }\,\left(\mathrm{mg}/\mathrm{dL}\right)-\mathrm{HDL }\left(\mathrm{mg}/\mathrm{dL}\right)-\frac{\mathrm{Triglyceride}}{5} \left(\mathrm{mg}/\mathrm{dL}\right)$$

Fasting blood was obtained from the tail vein after 5-h fasting. Triglyceride and total cholesterol levels were determined using colorimetric assay kits (ERBA Mannheim, Mannheim, Germany). Plasma HDL level was analyzed using a commercial assay kit (Biovision, Inc, Milpitas, CA, USA). LDL level was estimated using Friedewald’s equation: (Friedewald et al. 1972).

#### Echocardiography

Echocardiography (GE vivid‐I, GE healthcare, Chicago, IL, USA) was used to assess left ventricular (LV) function. At the beginning, light anesthesia was induced using 2% isoflurane under 3 L/min of oxygen. An echocardiography probe was then placed on the chest. The LV papillary muscle was located to identify M‐mode echocardiographic images for systolic function. LV ejection fraction (LVEF) and %LV fractional shortening (%LVFS) were measured to evaluate LV systolic function. The diastolic function was also determined using the ratio of early (E) to late (A) ventricular filling velocity (E/A ratio) detected from the color doppler velocity in an apical four‐chamber view (Amput, et al. 2020a, b).

#### Heart rate variability (HRV)

To determine HRV, a lead-II electrocardiogram was performed using Power Lab 4/25 T (AD Instruments, Sydney, NSW, Australia), and data was fed through a Chart 5.0 program (AD Instruments, Sydney, NSW, Australia). The HRV data were then analyzed using the MATLAB program. The parasympathetic tone was indicated by a high frequency (HF) at the range of 0.15–0.40 Hz. A low frequency (LF) at the range of 0.04–0.15 Hz was also identified as being representative of both parasympathetic and sympathetic tones. The cardiac autonomic balance was evaluated by the LF/HF ratio. A high LF/HF ratio indicated a cardiac sympathovagal imbalance (Chattipakorn et al. 2007).

#### Cardiac malondialdehyde (MDA) concentration

MDA concentration was quantified using high-performance liquid chromatography (HPLC). Briefly, the heart tissue was homogenized in a buffer containing 4 mM NaH_2_PO_4_.2H_2_O and 1 mM phosphoric acid at pH 2.8. The homogenate was mixed with 10% trichloroacetic acid containing butylated hydroxytoluene, heated at 90 °C for 30 min, and then centrifuged at 6,000 rpm for 10 min. Five hundred µL of the supernatant were mixed with 1.5 mL of 0.44 M H_3_PO_4_ and 1 mL of 0.6% thiobarbituric acid solution, and then incubated at 90 °C for 30 min to achieve a pink-colored product – thiobarbituric acid reactive substances (TBARS). The solution was filtered through a syringe filter (polysulfone type membrane, pore size 0.45 µm, Whatman International, Maidstone, UK). The final solution was then injected through the HPLC system using a Water Spherosorb ODS2 type column (250 × 4.3 mm, 5 µm) and a mobile-phase solvent of 50 mM KH_2_PO_4_ in methanol with a flow rate of 1 mL/min. Detection was then carried out at a wavelength of 532 nm. Data were analyzed using the BDS software (BarSpec Ltd., Rehovot, Israel). TBARS concentration was calculated under the standard curve and reported as the equivalent concentration of MDA (Pintana et al. 2013).

#### Western blot for protein expression analyses

For extraction of total protein, the heart tissue was lysed with an extraction buffer containing 1% Nonidet P-40, 0.5% sodium deoxycholate, 0.1% SDS in 1X PBS, and 1X protease inhibitor cocktail (Merck, KGaA, Darmstadt, Germany). After 30 min of incubation on ice, the homogenate was centrifuged at 13,000 rpm for 10 min. Two hundred μL of total protein (1 mg/mL) were then mixed with 50 μL of loading buffer containing 5% mercaptoethanol, 0.05% bromophenol blue, 75 nM Tris–HCl, 2% SDS, and 10% glycerol with pH 6.8. The mixture was heated at 95 °C for 10 min, and then kept at −80 °C until analysis.

For each analysis, 16 µg of total protein was loaded into each lane of sodium dodecyl sulfate (SDS)-polyacrylamide gels. The gels were then transferred to a nitrocellulose membrane and blocked for 1 h with 5% non-fat dry milk or 5% bovine serum albumin in Tris-buffer saline (pH 7.4) containing 0.1% Tween 20. The membranes were then probed overnight at 4 ºC with the primary antibodies. After that, they were incubated for 1 h at room temperature with horseradish peroxidase-conjugated secondary antibodies. The proteins on the membranes were visualized using ClarityTM Western ECL Blotting Substrate (Bio-Rad Laboratories Ltd., Hercules, CA, USA) and scanned through the ChemiDoc™ Touch Gel Imaging System (Bio-Rad Laboratories Ltd., Hercules, CA, USA). Finally, the density of each western blot band was quantified using ImageJ analysis software (NIH, Bethesda, MA, USA). The information about antibodies for protein expression analysis is provided in Supplementary Table 1.

#### Cardiac apoptotic cells by TUNEL assay

The heart tissue was fixed with 4% paraformaldehyde for 24 h and cryoprotected in 30% sucrose in PBS at 4 °C until the sectioning process. The tissue was cryosectioned into a thickness of 4 μm. The sections were then stored overnight at room temperature, followed by incubation with CytoninTM for 30 min. The sections were then immersed in 1X TdT labelling buffer, and were then incubated with a labelling reaction mix at 37 °C for 1 h. Lastly, cover slips were lowered over the sections which were then incubated with fluorescent mounting and DAPI. Images were collected using a confocal microscope (Olympus FLUOVIEW FV3000, Japan). Apoptotic index was reported as the percentage of TUNEL-positive cells from at least 500 nuclei of cardiomyocytes at 400× magnification.

#### Cardiac mitochondrial function assay

The cardiac mitochondria were isolated by the differential centrifugation procedure described elsewhere (Palee et al. 2013) to enable the evaluation of mitochondrial function, including mitochondrial reactive oxygen species (ROS) production, mitochondrial membrane potential change, and mitochondrial swelling.

For determination of mitochondrial ROS production, the isolated mitochondrial protein (0.4 mg/mL) was incubated with 2 µM of dichlorofluorescein diacetate (DCFH-DA) fluorescent dye, both with and without 2 mM of H_2_O_2_, at 25 °C for 20 min. Fluorescence intensity was measured using a fluorescent microplate reader (Bio-Tek Instruments Inc., Winooski, VT, USA) with an excitation wavelength of 485 nm and an emission wavelength of 530 nm. The mitochondrial ROS production was reported as a fold increment of ROS after H_2_O_2_ stimulation.

Mitochondrial membrane potential change (ΔΨm) was measured using JC-1 fluorescent dye. The mitochondrial protein (0.4 mg/mL) was incubated with 310 nM of JC-1, both with and without 2 mM of H_2_O_2_, at 37 °C for 30 min. The excitation and emission wavelengths of the JC-1 monomer form that provide green fluorescence were 485 nm and 530 nm, respectively. In contrast, the excitation and emission wavelengths of the JC-1 monomer form that provide red fluorescence were 485 nm and 590 nm, respectively. The ∆ψm was calculated from the red/green intensity ratio using a fluorescent microplate reader (Bio-Tek Instruments, Inc., Winooski, VT, USA). Greater mitochondrial depolarization was indicated by a greater reduction in this ratio after H_2_O_2_ stimulation.

Cardiac mitochondrial swelling was measured by the dynamic change in absorbance of the mitochondrial protein (0.4 mg/mL) at a wavelength of 540 nm using a microplate reader (Bio-Tek Instruments, Winooski, VT, USA). The absorbance at 30 min was normalized to the absorbance at baseline and presented as a ratio. A lower ratio indicates a greater degree of mitochondrial swelling.

#### Cardiac mitochondrial respiration assay

The cardiac mitochondria were isolated by the differential centrifugation procedure as detailed in a prior study (Palee et al. 2013). The isolate was used to measure mitochondrial respiration via a high-throughput automated 96-well extracellular flux analyzer (Agilent seahorse XFe96, Santa Clara, CA, USA). Isolated cardiac mitochondrial protein (0.4 mg/mL) was suspended in a medium buffer containing 100 mM KCl, 10 mM HEPES, and 5 mM KH_2_PO_4_ at pH 7.2 in the presence of 0.2% fatty acid-free bovine serum albumin. The XFe96 plate was then coated with polyethylenimine (1:15,000 dilution), and incubated overnight at 37 °C. The polyethylenimine was removed on the day of the assay, and then 20 µL of cardiac mitochondrial protein was loaded into the XFe96 plate, and was centrifuged at 3,000 g for 7 min at 4 °C. The mitochondrial protein was then incubated for 30 min at 37 °C before the beginning of the assay. During the assay, 5 mM pyruvate and 5 mM malate were added first as NADH-linked substrates, followed by 1 mM of ADP administration to determine state-3 mitochondrial respiration. State 4 respiration was then measured after adding 1 µM of oligomycin. The respiratory control ratio was calculated from the state 3/state 4 respiration ratio (Arinno et al. 2019).

#### Statistical analyses

For the comparison between normal and PDM groups at week 12 of the experiment, data were analyzed using unpaired two-tail Student’s t-tests. The data from the normal, PDM, PDMCR-SM, PDMCR-L, PDMEX-SM, and PDMEX-L groups at week 28 of the experiment were compared using a one-way ANOVA followed by Fisher's least significant difference analysis. Correlations within the same group were identified using Pearson’s correlation coefficient. A p-value < 0.05 was considered as a measure of statistical significance.

## Results

Both caloric restriction and exercise improved insulin sensitivity and lipid profiles. Long-term caloric restriction exhibited the greatest benefits, compared to other interventions in prediabetic rats.

After 12 weeks of HFD consumption, the rats had increased fasting glucose, fasting insulin, and HOMA-IR, when compared to those of ND rats (Table 1). However, the levels of fasting glucose of HFD-fed rats were less than 300 mg/dL (183.82 ± 5.64 mg/dL). All of these results suggested that these HFD-rats developed prediabetes (Domon et al. 2019). At week 28 of the experiment, our HFD-fed rats remained prediabetic with a fasting glucose level of 228.86 ± 15.70 mg/dL (Table 1). In prediabetic rats in the groups of short-term caloric restriction followed by weight maintenance, short-term exercise followed by weight maintenance, and long-term exercise the fasting glucose levels had been restored to normal to a similar extent (Table 1). Rats with long-term caloric restriction had even further reduction in fasting glucose level which was significantly lower than that of ND rats. With regard to the insulin level, all of the interventions effectively reduced the fasting insulin level back to the normal level in prediabetic rats. In addition, complete restoration of HOMA-IR to the normal level was observed in the prediabetic rats receiving short-term caloric restriction followed by weight maintenance, short-term exercise followed by weight maintenance, and long-term exercise. The prediabetic rats in the long-term caloric restriction group demonstrated the lowest level of HOMA-IR among all groups (Table 1).

**Table 1:** Insulin resistance profile and lipid profile in the blood

Parameters	Week 12		Week 28					
	**ND** **(n = 6)**	**PDM** **(n = 30)**	**ND** **(n = 6)**	**PDM** **(n = 6)**	**PDMCR-SM** **(n = 6)**	**PDMCR-L** **(n = 6)**	**PDMEX-SM** **(n = 6)**	**PDMEX-L** **(n = 6)**
Fasting glucose (mg/dL)	107.90 ± 6.35	183.82 ± 5.64^*^	183.45 ± 7.82	228.86 ± 15.70^*^	164.88 ± 12.56^†^	143.54 ± 6.44^*†^	183.15 ± 19.67^†§^	186.89 ± 4.32^†§^
Fasting insulin (mU/mL)	4.55 ± 0.67	7.25 ± 0.64^*^	5.55 ± 0.84	9.60 ± 1.29^*^	5.32 ± 0.57^†^	5.45 ± 1.02^†^	5.81 ± 1.55^†^	6.02 ± 1.08^†^
HOMA-IR	1.24 ± 0.23	3.09 ± 0.32^*^	3.38 ± 0.39	5.68 ± 0.68^*^	2.51 ± 0.23^†^	1.59 ± 0.21^*†^	3.41 ± 0.90^†§^	3.39 ± 0.67^†§^
Triglyceride (mg/dL)	56.99 ± 8.72	84.37 ± 5.50^*^	121.11 ± 11.21	199.58 ± 19.92^*^	123.65 ± 12.68^†^	78.21 ± 5.12^*†‡^	123.50 ± 9.53^†§^	122.92 ± 12.69^†§^
Total cholesterol (mg/dL)	74.15 ± 5.52	98.78 ± 4.65^*^	98.52 ± 8.52	132.4 ± 9.03^*^	99.43 ± 806^†^	98.00 ± 4.80^†^	96.61 ± 8.95^†^	98.06 ± 8.74^†^
HDL (mg/dL)	29.64 ± 1.29	22.40 ± 0.98^*^	33.41 ± 1.40	23.98 ± 2.27^*^	34.57 ± 1.97^†^	34.07 ± 2.64^†^	33.90 ± 2.60^†^	34.12 ± 2.65^†^
Calculated LDL (mg/dL)	29.68 ± 3.58	53.90 ± 3.56^*^	46.49 ± 8.89	73.90 ± 3.53^*^	46.58 ± 7.30^†^	45.69 ± 1.36^†^	43.03 ± 7.89^†^	41.45 ± 9.03^†^

Data are reported as mean ± SEM

^*^p < 0.05 when compared to ND, ^†^p < 0.05 when compared to PDM, ^‡^p < 0.05 when compared to PDMCR-SM, ^§^p < 0.05 when compared to PDMCR-L, ^*‖*^p < 0.05 when compared to PDMEX-SM

ND: Normal diet; PDM: Prediabetes with no intervention; PDMCR-SM: Prediabetes with short-term caloric restriction followed by weight maintenance; PDMCR-L: Prediabetes with long-term caloric restriction; PDMEX-SM: Prediabetes with short-term exercise followed by weight maintenance; PDMEX-L: Prediabetes with long-term exercise

Triglyceride, total cholesterol, and LDL levels were greater, but HDL levels were lower in prediabetic rats, when compared with those of ND rats (Table 1). These results indicated that prediabetic rats developed dyslipidemia. Short-term caloric restriction followed by weight maintenance, long-term caloric restriction, short-term exercise followed by weight maintenance, and long-term exercise restores levels of total cholesterol, LDL, and HDL to normal in these prediabetic rats. Considering triglyceride, short-term caloric restriction followed by weight maintenance, short-term exercise followed by weight maintenance, and long-term exercise could decrease triglyceride levels to within normal limits, while long-term caloric restriction reduced triglyceride levels even further to the lowest among all groups (Table 1).

Both caloric restriction and exercise resulted in a decrease in body weight and visceral fat weight. The long-term caloric restriction exerted the greatest benefits, compared with other interventions in prediabetic rats.

**Fig. 2:**
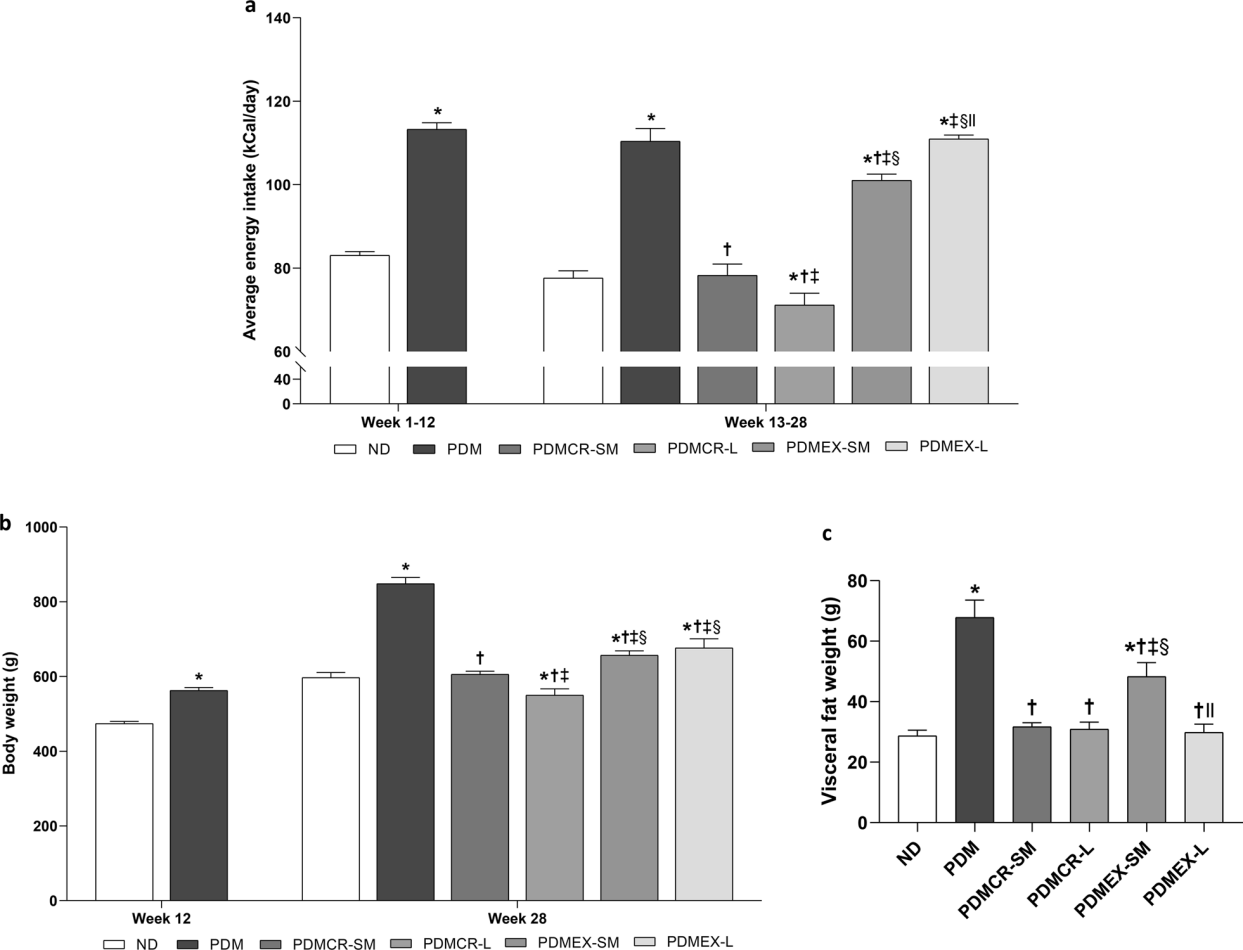
Average energy intake (**a**), Body weight (**b**), Visceral fat weight (**c**). n = 5–6 per group. Data are reported as mean ± SEM. *p < 0.05 when compared to ND, ^†^p < 0.05 when compared to PDM, ^‡^p < 0.05 when compared to PDMCR-SM, ^§^p < 0.05 when compared to PDMCR-L, ^‖^p < 0.05 when compared to PDMEX-SM. ND: Normal diet; PDM: Prediabetes with no intervention; PDMCR-SM: Prediabetes with short-term caloric restriction followed by weight maintenance; PDMCR-L: Prediabetes with long-term caloric restriction; PDMEX-SM: Prediabetes with short-term exercise followed by weight maintenance; PDMEX-L: Prediabetes with long-term exercise

Due to their consumption of a HFD, prediabetic rats consumed a higher energy intake than ND rats. During the interventions from weeks 13–28, long-term caloric restriction resulted in the lowest energy intake among all interventions (Fig. 2a). However, energy intake of ND rats and prediabetic rats in the short-term caloric restriction followed by weight maintenance group was no different. In the case of the rats in the exercise groups, the energy intake of rats having short-term exercise followed by weight maintenance, who consumed HFD followed by ND, was higher than that of ND rats, but was still lower than that of prediabetic rats with no intervention and with long-term exercise who consumed only HFD (Fig. 2a).

Regarding body weight, HFD consumption led to a heavier body weight in prediabetic rats. Long-term caloric restriction resulted in the lowest body weight among all interventions (Fig. 2b). Body weight of ND rats and prediabetic rats following the short-term caloric restriction regimen followed by weight maintenance was no different. In the exercise rats, the body weight of prediabetic rats on the short-term exercise program followed by weight maintenance and prediabetic rats having long-term exercise was no different either. However, both groups of exercised rats weighed heavier than normal-diet rats, but were still lighter than prediabetic rats with no intervention (Fig. 2b).

Consistent with the body weight, the visceral fat weight of prediabetic rats were heaviest among all groups. Interestingly, short-term caloric restriction followed by weight maintenance, long-term caloric restriction, and long-term exercise led to a reduction of visceral fat weight to a similar extent, and this became no different to that of the ND rats (Fig. 2c). On the other hand, the visceral fat weight of prediabetic rats having short-term exercise followed by weight maintenance remained higher than that of ND rats (Fig. 2c).

### Exercise was the only intervention to improve whole-body substrate oxidation rates during vigorous exercise in prediabetic rats

**Fig. 3:**
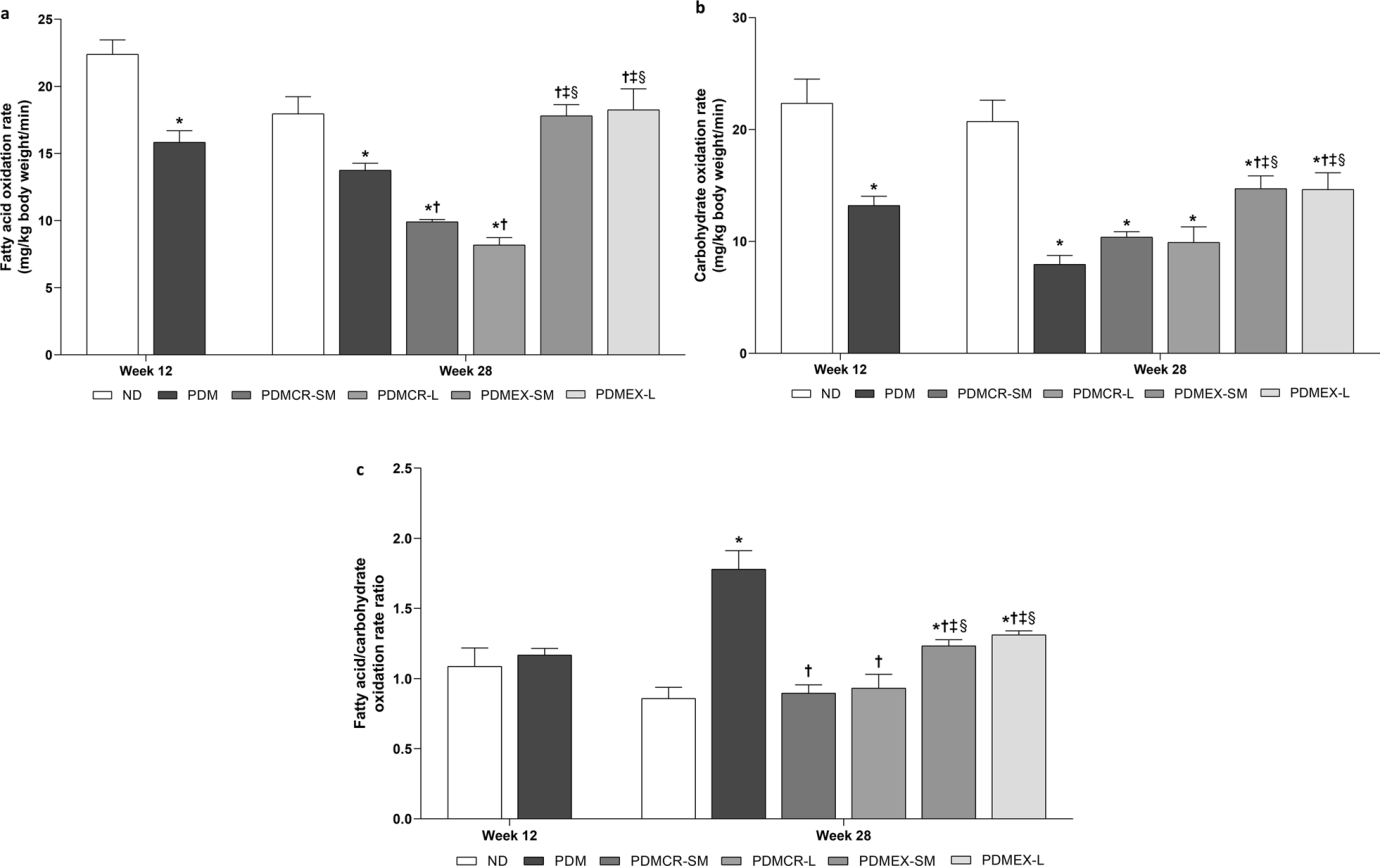
Fatty acid oxidation rate (**a**), Carbohydrate oxidation rate (**b**), Fatty acid/carbohydrate oxidation rate ratio (**c**). n = 5–6 per group. Data are reported as mean ± SEM. *p < 0.05 when compared to ND, ^†^p < 0.05 when compared to PDM, ^‡^p < 0.05 when compared to PDMCR-SM, ^§^p < 0.05 when compared to PDMCR-L, ^‖^p < 0.05 when compared to PDMEX-SM. ND: Normal diet; PDM: Prediabetes with no intervention; PDMCR-SM: Prediabetes with short-term caloric restriction followed by weight maintenance; PDMCR-L: Prediabetes with long-term caloric restriction; PDMEX-SM: Prediabetes with short-term exercise followed by weight maintenance; PDMEX-L: Prediabetes with long-term exercise

Whole-body substrate oxidation rates during vigorous exercise were measured using indirect calorimetry. These included fatty acid oxidation rate and carbohydrate oxidation rate. When compared with ND rats, fatty acid oxidation rates were lower in prediabetic rats (Fig. 3a). Our results showed that short-term exercise followed by weight maintenance and long-term exercise led to the restoration of fatty acid oxidation rate back to within normal limits. Interestingly, short-term caloric restriction followed by weight maintenance and long-term caloric restriction resulted in a further reduction of fatty acid oxidation rate in prediabetic rats (Fig. 3a).

Consistent with the fatty acid oxidation rate, the carbohydrate oxidation rate was lower in prediabetic rats than those of ND rats (Fig. 3b). Short-term exercise followed by weight maintenance and long-term exercise did lead to an increase in the carbohydrate oxidation rate, but the levels remained lower than that of ND rats. In contrast, both the short-term caloric restriction followed by weight maintenance and the long-term caloric restriction failed to increase the carbohydrate oxidation rate in prediabetic rats (Fig. 3b).

To determine the preference of fat utilization as a fuel source, the fatty acid/carbohydrate oxidation rate ratio was calculated. Our results demonstrated that this ratio was no different between ND and prediabetic rats at week 12 of the experiment. However, this ratio became much higher in prediabetic rats than that of ND rats at week 28 of the experiment. The short-term caloric restriction followed by weight maintenance and the long-term caloric restriction did reduce this ratio back to normal. However, the short-term exercise followed by weight maintenance and the long-term exercise only partially decreased this ratio in prediabetic rats (Fig. 3c).

Both caloric restriction and exercise improved cardiac function. The short-term exercise followed by weight maintenance had the least benefit on the improvement of diastolic function, compared to other interventions in prediabetic rats.

**Fig. 4:**
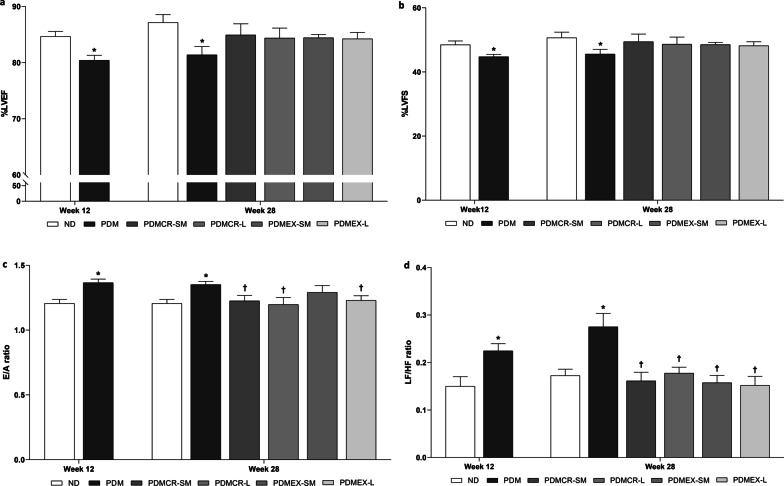
%LVEF (**a**), %LVFS (**b**), E/A ratio (**c**), LF/HF ratio (**d**). n = 5–6 per group. Data are reported as mean ± SEM. *p < 0.05 when compared to ND, ^†^p < 0.05 when compared to PDM, ^‡^p < 0.05 when compared to PDMCR-SM, ^§^p < 0.05 when compared to PDMCR-L, ^‖^p < 0.05 when compared to PDMEX-SM. ND: Normal diet; PDM: Prediabetes with no intervention; PDMCR-SM: Prediabetes with short-term caloric restriction followed by weight maintenance; PDMCR-L: Prediabetes with long-term caloric restriction; PDMEX-SM: Prediabetes with short-term exercise followed by weight maintenance; PDMEX-L: Prediabetes with long-term exercise

In comparison with ND rats, a decrease in %LVEF and %LVFS, along with an increase in the E/A ratio and the LF/HF ratio were observed in prediabetic rats, indicating impaired systolic, diastolic, and cardiac autonomic function, respectively (Fig. 4a-d). All intervention regimens in this protocol led to the restoration of %LVEF, %LVFS, and the LF/HF ratio to normal. Short-term caloric restriction followed by weight maintenance, long-term caloric restriction, and long-term exercise caused the reduction of the E/A ratio back to normal, whereas the short-term exercise followed by weight maintenance could just alleviate an increase in E/A ratio in the prediabetic rats (Fig. 4a-d).

### Both caloric restriction and exercise regimens improve cardiac mitochondrial function and oxidative phosphorylation in prediabetic rats

**Fig. 5:**
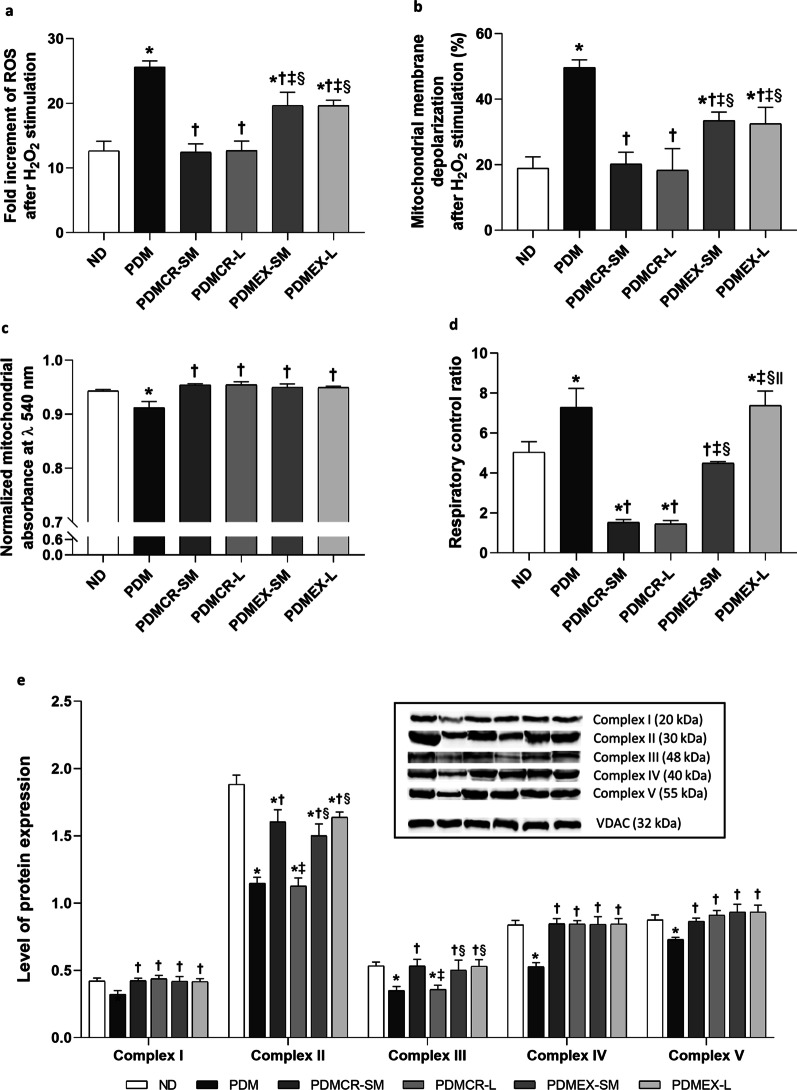
Cardiac mitochondrial ROS production (**a**), Cardiac mitochondrial membrane depolarization (**b**), Cardiac mitochondrial swelling indicated by the normalized absorbance at λ 540 nm (**c**), Cardiac respiratory control ratio (**d**), Cardiac OXPHOS protein expression (**e**). VDAC was used as a housekeeping protein. n = 5–6 per group. Data are reported as mean ± SEM. *p < 0.05 when compared to ND, ^†^p < 0.05 when compared to PDM, ^‡^p < 0.05 when compared to PDMCR-SM, ^§^p < 0.05 when compared to PDMCR-L, ^‖^p < 0.05 when compared to PDMEX-SM. ND: Normal diet; PDM: Prediabetes with no intervention; PDMCR-SM: Prediabetes with short-term caloric restriction followed by weight maintenance; PDMCR-L: Prediabetes with long-term caloric restriction; PDMEX-SM: Prediabetes with short-term exercise followed by weight maintenance; PDMEX-L: Prediabetes with long-term exercise

Cardiac mitochondrial function was assessed by examining mitochondrial ROS production, mitochondrial membrane depolarization, and mitochondrial swelling (Fig. 5a–c). Prediabetic rat hearts had an increase in mitochondrial ROS production, mitochondrial membrane depolarization, and mitochondrial swelling, as indicated by a decrease in normalized mitochondrial absorbance (Fig. 5a–c). Short-term caloric restriction followed by weight maintenance and long-tern caloric restriction resulted in a decrease in mitochondrial ROS production and mitochondrial membrane depolarization back to normal, whereas the short-term exercise program followed by weight maintenance and long-term exercise could just ameliorate these abnormalities in prediabetic rats (Fig. 5a, b). With regard to mitochondrial swelling, all of these interventions could effectively decrease mitochondrial swelling back to normal (Fig. 5c).

Mitochondrial respiration was determined by respiratory control ratio. The results demonstrated that prediabetic rats with no intervention and with long-term exercise exhibited significantly higher levels of the respiratory control ratio than that of ND rats (Fig. 5d). In contrast, prediabetic rats with the short-term caloric restriction followed by weight maintenance and those with long-term caloric restriction had significantly lower levels of respiratory control ratio, when compared with that of ND rats. However, the respiratory control ratio of ND rats and prediabetic rats on the short-term exercise program followed by weight maintenance was no different (Fig. 5d).

Cardiac mitochondrial oxidative phosphorylation was also evaluated. Prediabetic rats had decreased oxidative phosphorylation complex content, as indicated by a reduction of complexes I-V protein expressions (Fig. 5e and Additional file 1: Fig. S1). The rats in the short-term caloric restriction followed by weight maintenance, the long-term caloric restriction, the short-term exercise followed by weight maintenance, and the long-term exercise groups showed an increase in the expression of complexes I, IV, and V back to normal (Fig. 5e and Additional file 1: Fig. S1).

### Although both caloric restriction and exercise improve cardiac insulin signaling, long-term exercise is the only intervention to improve cardiac metabolism and mitochondrial biogenesis in prediabetic rats

**Fig. 6:**
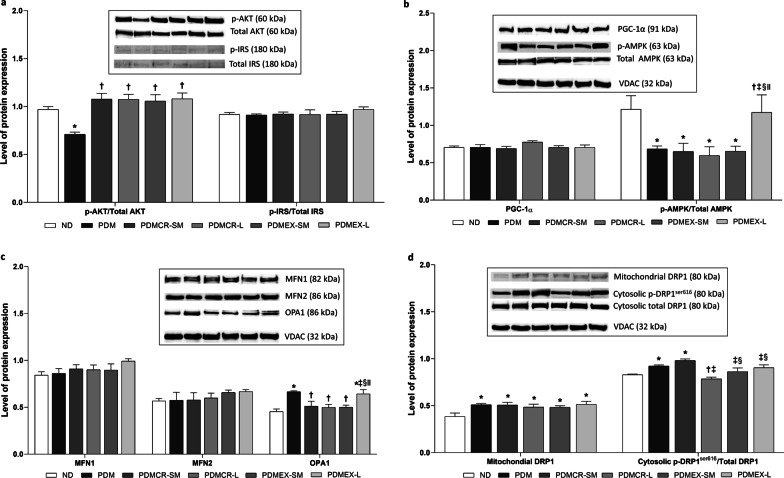
Expression of insulin signaling-related proteins in the heart (**a**), Expression of mitochondrial biogenesis and metabolism-related proteins in the heart (**b**), Expression of mitochondrial fusion-related proteins in the heart (**c**), Expression of mitochondrial fission-related proteins in the heart (**d**). VDAC was used as a housekeeping protein. n = 5–6 per group. Data are reported as mean ± SEM. *p < 0.05 when compared to ND, ^†^p < 0.05 when compared to PDM, ^‡^p < 0.05 when compared to PDMCR-SM, ^§^p < 0.05 when compared to PDMCR-L, ^‖^p < 0.05 when compared to PDMEX-SM. ND: Normal diet; PDM: Prediabetes with no intervention; PDMCR-SM: Prediabetes with short-term caloric restriction followed by weight maintenance; PDMCR-L: Prediabetes with long-term caloric restriction; PDMEX-SM: Prediabetes with short-term exercise followed by weight maintenance; PDMEX-L: Prediabetes with long-term exercise

Our results showed that p-AKT/total AKT protein expression was lower in prediabetic rats, when compared to that of ND rats. This expression increased back to normal following all types of interventions, including the short-term caloric restriction followed by weight maintenance, the long-term caloric restriction, the short-term exercise followed by weight maintenance, and the long-term exercise (Fig. 6a and Additional file 1: Fig. S2). However, p-IRS/total IRS protein expression, which is also involved in insulin signaling pathway, was no different between all groups of rats (Fig. 6a and Additional file 1: Fig. S2).

Our results also showed that p-AMPK/total AMPK protein expression was lower in prediabetic rats than that in ND rats. Interestingly, only the rats in the long-term exercise group had expression of p-AMPK/total AMPK protein restored to within normal limits, whereas other interventions did not attenuate the reduction of this protein expression in prediabetic rats (Fig. 6b and Additional file 1: Fig. S2). The level of PGC-1α protein expression was no different between all groups of rats (Fig. 6b and Additional file 1: Fig. S2).

### Long-term caloric restriction resulted in the greatest benefit as regards the attenuation of cardiac mitochondrial fission, compared to other interventions in prediabetic rats

In the case of cardiac mitochondrial fusion, the expression of MFN1 and MFN2 proteins were no different between all groups of rats (Fig. 6c and Additional file 1: Fig. S3). Consistent with the findings regarding the respiratory control ratio, the expression of OPA1 was greater in prediabetic rats with no intervention and those doing the long-term exercise, when compared with that of other groups of rats (Fig. 6c and Additional file 1: Fig. S3).

As regards cardiac mitochondrial fission, the expression of mitochondrial DRP1 and cytosolic p-DRP1^ser616^/total DRP1 proteins was greater in prediabetic rats than those of ND rats, suggesting prediabetes induced cardiac mitochondrial fission (Fig. 6d and Additional file 1: Fig. S3). Long-term caloric restriction, short-term exercise followed by weight maintenance, and long-term exercise resulted in a decrease in cytosolic p-DRP1^ser616^/total DRP1 protein expression back to normal. The long-term caloric restriction exerted the greatest reduction in this protein expression among these three interventions (Fig. 6d and Additional file 1: Fig. S3).

### Long-term caloric restriction was the only intervention to decrease cardiac apoptosis to within normal limits in prediabetic rats

**Fig. 7:**
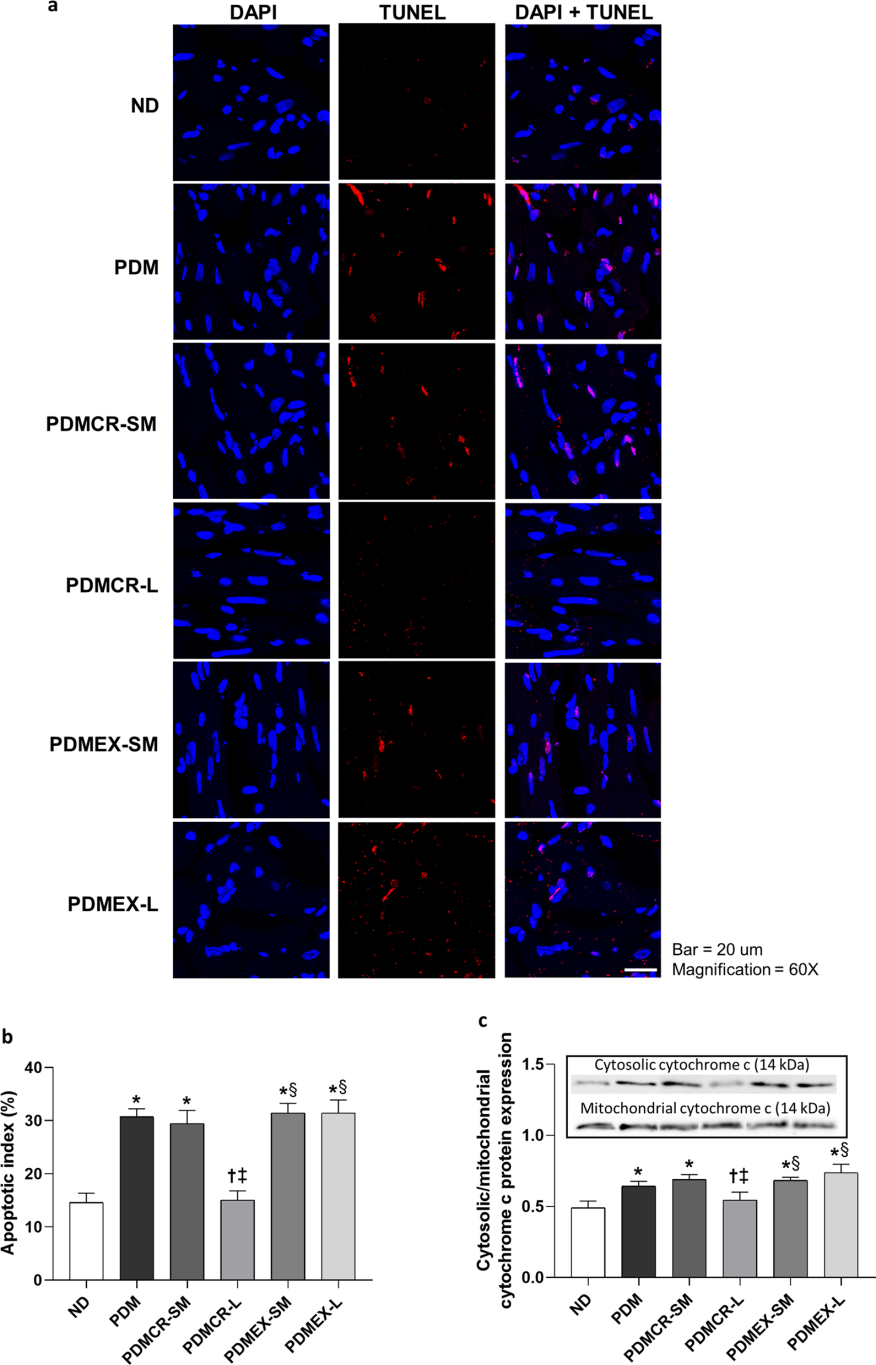
TUNEL assay representative of cardiac apoptosis from a rat of each group (**a**), Apoptotic index in the heart (**b**), Expression of cytosolic/mitochondrial cytochrome protein in the heart (**c**). n = 5–6 per group. Data are reported as mean ± SEM. *p < 0.05 when compared to ND, ^†^p < 0.05 when compared to PDM, ^‡^p < 0.05 when compared to PDMCR-SM, ^§^p < 0.05 when compared to PDMCR-L, ^‖^p < 0.05 when compared to PDMEX-SM. ND: Normal diet; PDM: Prediabetes with no intervention; PDMCR-SM: Prediabetes with short-term caloric restriction followed by weight maintenance; PDMCR-L: Prediabetes with long-term caloric restriction; PDMEX-SM: Prediabetes with short-term exercise followed by weight maintenance; PDMEX-L: Prediabetes with long-term exercise

We observed that cytosolic/mitochondrial cytochrome c protein expression and apoptotic index were higher in prediabetic rats than that in ND rats, indicating prediabetes induced cardiac apoptosis. Interestingly, only the rats in the long-term caloric restriction group had restored levels of cytosolic/mitochondrial cytochrome c protein expression and apoptotic index back to the normal level (Fig. 7a-c and Additional file 1: Fig. S4).

### Exercise was the only intervention to decrease cardiac lipid peroxidation, and only long-term interventions reduced cardiac inflammation back to normal in prediabetic rats

**Fig. 8:**
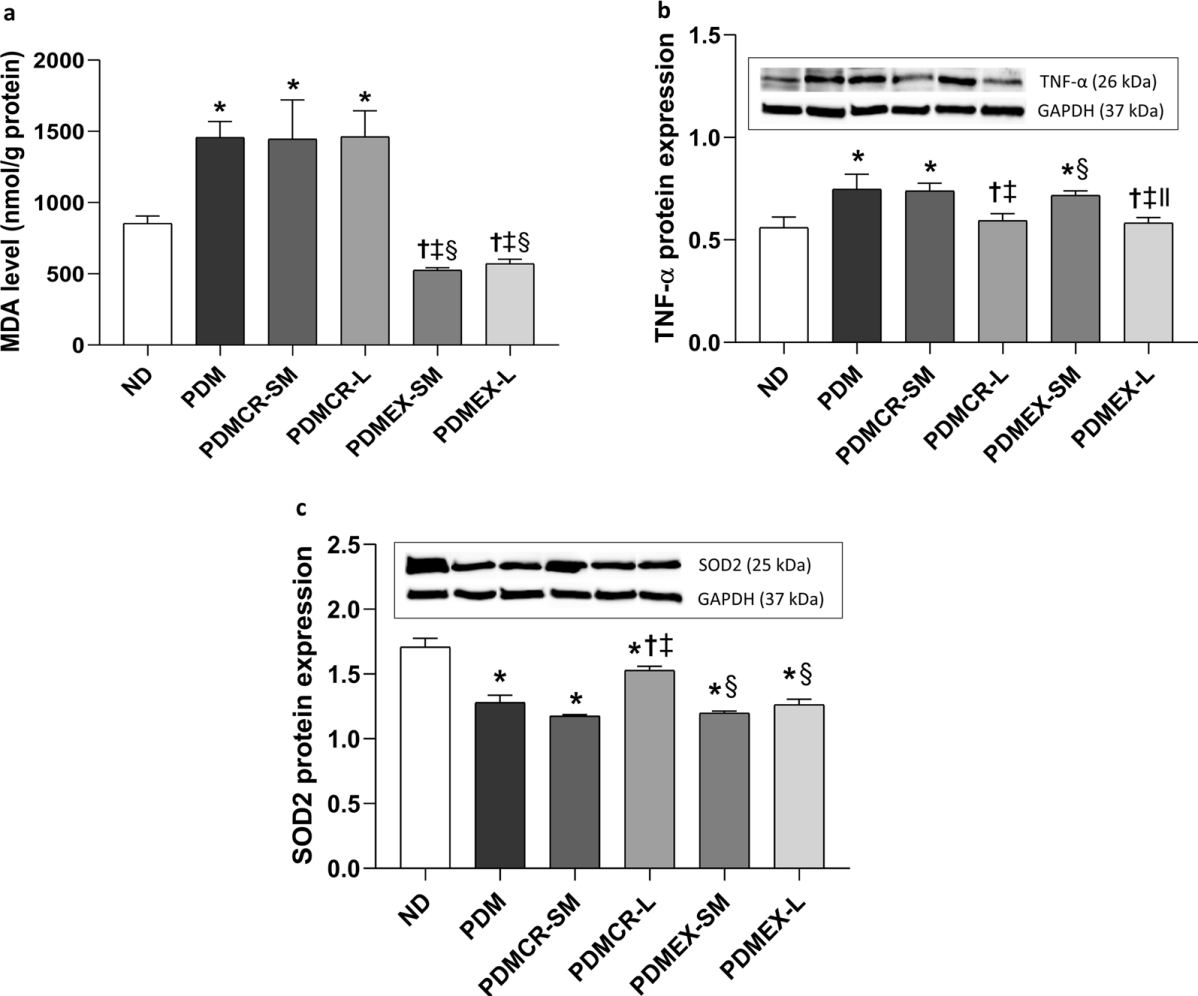
Cardiac MDA level (**a**), Expression of TNF-α protein in the heart (**b**), Expression of SOD2 protein in the heart (**c**). GAPDH was used as a housekeeping protein. n = 5–6 per group. Data are reported as mean ± SEM. *p < 0.05 when compared to ND, ^†^p < 0.05 when compared to PDM, ^‡^p < 0.05 when compared to PDMCR-SM, ^§^p < 0.05 when compared to PDMCR-L, ^‖^p < 0.05 when compared to PDMEX-SM. ND: Normal diet; PDM: Prediabetes with no intervention; PDMCR-SM: Prediabetes with short-term caloric restriction followed by weight maintenance; PDMCR-L: Prediabetes with long-term caloric restriction; PDMEX-SM: Prediabetes with short-term exercise followed by weight maintenance; PDMEX-L: Prediabetes with long-term exercise

The cardiac MDA level was higher in prediabetic rats than that in ND rats, suggesting prediabetes induced cardiac lipid peroxidation. Only rats in the exercise groups (i.e., both the short-term exercise followed by weight maintenance and the long-term exercise) showed a decrease in the MDA level back to normal limits in prediabetic rats (Fig. 8a).

Regarding cardiac inflammation, our results revealed that TNF-α protein expression was greater in prediabetic rats, when compared with that of ND rats. Importantly, TNF-α protein expression could decrease back to normal after long-term caloric restriction and long-term exercise. On the other hand, in the short-term caloric restriction followed by weight maintenance and the short-term exercise followed by weight maintenance groups the level of TNF-α protein expression in prediabetic rats did not decrease (Fig. 8b and Additional file 1: Fig. S4).

### Prediabetes resulted in diminished antioxidative capacity, which could only be improved by long-term caloric restriction out of these interventions

Our results showed that SOD2 protein expression was lower in prediabetic rats than that of ND rats. Long-term caloric restriction could partially increase SOD2 protein expression in prediabetic rats, whereas in the short-term caloric restriction followed by weight maintenance, the short-term exercise followed by weight maintenance, and the long-term exercise groups there was not change in this value (Fig. 8c and Additional file 1: Fig. S4).

### Both caloric restriction and exercise improved CRF level. The long-term caloric restriction exerted the greatest benefit, compared with other interventions in prediabetic rats

**Fig. 9:**
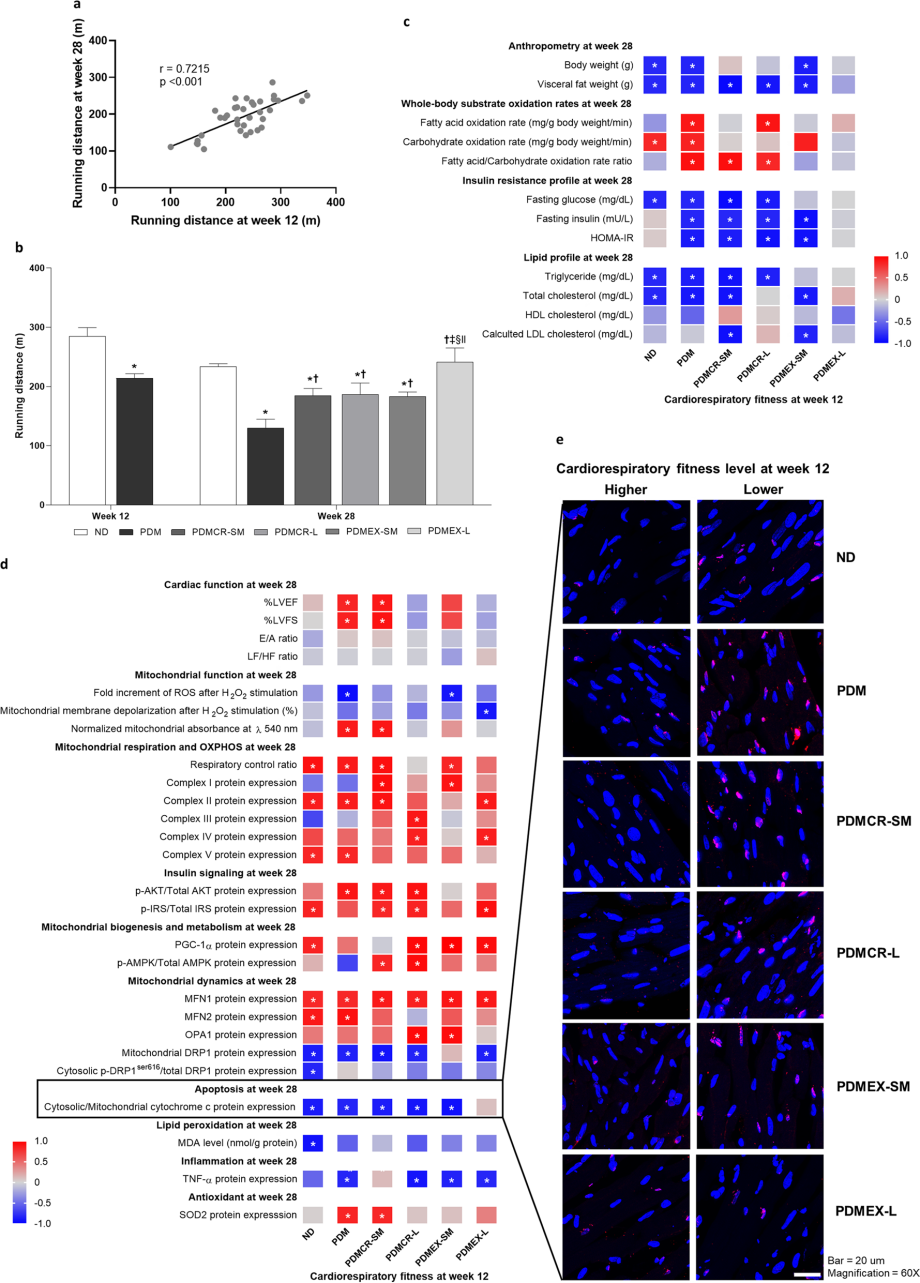
The correlation between running distance at week 12 and week 28 (**a**), Running distance values (**b**), The correlations between CRF level at baseline (week 12) versus anthropometry and metabolic parameters at follow-up (week 28) within each group of rats (**c**), The correlations between CRF level at baseline (week 12) and cardiac parameters at follow-up (week 28) within each group of rats (**d**), TUNEL assay representative of cardiac apoptosis between high-CRF level and low-CRF level rats in each group (**e**). CRF was reported as running distance. n = 5–6 per group. Data are reported as mean. *p < 0.05. ND: Normal diet; PDM: Prediabetes with no intervention; PDMCR-SM: Prediabetes with short-term caloric restriction followed by weight maintenance; PDMCR-L: Prediabetes with long-term caloric restriction; PDMEX-SM: Prediabetes with short-term exercise followed by weight maintenance; PDMEX-L: Prediabetes with long-term exercise

In this study, CRF was measured and reported as the running distance value. We calculated the correlation between running distance at baseline (week 12 of the experiment) and at follow-up (week 28 of the experiment). There was a significant positive correlation between running distance at baseline and at follow-up (Fig. 9a). Our results showed that the running distance was lower in prediabetic rats than that of ND rats. The long-term exercise restored the running distance to normal. However, the short-term caloric restriction followed by weight maintenance, the long-term caloric restriction, and the short-term exercise followed by weight maintenance interventions only led to a partial increase in running distance (Fig. 9b).

### High CRF exerts a protection against severe metabolic syndrome in prediabetic rats, both with and without caloric restriction or short-term exercise followed by weight maintenance

To clarify the effects of high CRF on conferring protection against severe metabolic syndrome in prediabetic rats, we investigated the correlations between CRF level at baseline (week 12 of the experiment) versus anthropometry and metabolic parameters at follow-up (week 28 of the experiment) within each group of rats.

In ND rats, there were negative correlations between the baseline CRF level versus body weight, visceral fat weight, fasting glucose, triglyceride, and total cholesterol at follow-up, whereas a positive correlation between the baseline CRF level and the carbohydrate oxidation rate at follow-up was observed (Fig. 9c).

In prediabetic rats with no intervention, negative correlations between CRF level at baseline versus body weight, visceral fat weight, fasting glucose, fasting insulin, HOMA-IR, triglyceride, and total cholesterol at follow-up were demonstrated, whereas there were positive correlations between carbohydrate oxidation rate, fatty acid oxidation rate, and fatty acid/carbohydrate oxidation rate ratio at follow-up (Fig. 9c). All of these results indicated that high CRF also exerts protection against severe metabolic syndrome in prediabetic rats.

Consistent with ND rats and prediabetic rats with no intervention, high baseline CRF level was also significantly correlated with favorable anthropometry, whole-body substrate oxidation rates, insulin sensitivity profile, and lipid profile at follow-up in prediabetic rats receiving short-term caloric restriction followed by weight maintenance, long-term caloric restriction, and short-term exercise followed by weight maintenance. However, these significant correlations were not observed in prediabetic rats having long-term exercise (Fig. 9c). All of these results suggested that high CRF remains protective against severe metabolic syndrome in prediabetic rats having caloric restriction or short-term exercise followed by weight maintenance, but not in those receiving long-term exercise.

### High CRF exerted protection against cardiac impairment in prediabetic rats, both with and without lifestyle modification

To elucidate the protective effects of high CRF against cardiac impairment in prediabetic rats, we evaluated the correlations between CRF level at baseline (week 12 of the experiment) versus cardiac parameters at follow-up (week 28 of the experiment) within each group of rats.

In ND rats, there were negative correlations between baseline CRF level and mitochondrial DRP1 protein expression, cytosolic p-DRP1^ser616^/total DRP1 protein expression, cytosolic/mitochondrial cytochrome c protein expression, and MDA level at follow-up. In contrast, positive correlations were shown between baseline CRF level and p-IRS/total IRS protein expression, PGC-1α protein expression, respiratory control ratio, complex II and V protein expressions, MFN1 protein expression, and MFN2 protein expression at follow-up (Fig. 9d, e). All of these results suggested that high CRF is protective against cardiac impairment via the modulation of insulin signaling, mitochondrial biogenesis, metabolism, mitochondrial respiration, oxidative phosphorylation, mitochondrial dynamics, apoptosis, and lipid peroxidation.

In prediabetic rats, we found negative correlations between CRF level at baseline and mitochondrial ROS production, mitochondrial swelling, mitochondrial DRP1 protein expression, cytosolic/mitochondrial cytochrome c protein expression, and TNF-α protein expression at follow-up. However, there were positive correlations between CRF level at baseline and %LVEF, %LVFS, p-AKT/total AKT protein expression, respiratory control ratio, complex II and V protein expressions, MFN1 protein expression, MFN2 protein expression, and SOD2 protein expression at follow-up (Fig. 9d, e). All of these findings suggested that the high CRF also exerts protection against cardiac impairment in prediabetes. This effect appears to be mediated by the modulation of mitochondrial function, insulin signaling, metabolism, mitochondrial respiration, oxidative phosphorylation, mitochondrial dynamics, apoptosis, antioxidative capacity, inflammation, and systolic function.

Consistent with the findings from ND rats and prediabetic rats with no intervention, high CRF level at baseline also showed a significant correlation with favorable cardiac mitochondrial health, cardiac insulin sensitivity, decreased cardiac apoptosis, and decreased cardiac inflammation at follow-up in prediabetic rats in the short-term caloric restriction followed by weight maintenance, long-term caloric restriction, short-term exercise followed by weight maintenance, and long-term exercise groups (Fig. 9d, e). All of these results suggested that high CRF remains protective against severe cardiac impairment in prediabetic rats with lifestyle interventions.

## Discussion

With regard to blood metabolic parameters, the interventions short-term caloric restriction followed by weight maintenance, short-term exercise followed by weight maintenance, and long-term exercise brought insulin sensitivity and lipid levels back to normal in prediabetic rats. These results emphasized that short-term interventions followed by weight maintenance also exert the measurable therapeutic effects on prediabetes-induced metabolic impairment. However, long-term caloric restriction decreased fasting glucose, HOMA-IR, and triglyceride levels even further to levels lower than those of ND rats of the same age. These findings are likely to be the result of lower body weight in our prediabetic rats with long-term caloric restriction, as compared with that of our ND rats (Anderson et al. 2001). Since higher levels of fasting glucose, HOMA-IR, and triglyceride are associated with increased risk of all-cause and cardiovascular mortality in the general population (Liu et al. 2020), our results supported the theory that long-term caloric restriction promotes longevity even in non-obese and non-prediabetic conditions (Pifferi and Aujard 2019).

Insulin plays an important role in glucose oxidation and suppression of lipolysis (Chakrabarti et al. 2013; Gaster and Beck-Nielsen 2004; Hickner et al. 1999; Karwi et al. 2020). A reduction in carbohydrate oxidation rate with an increase in fatty acid/carbohydrate oxidation rate ratio in our prediabetic rats suggested the development of insulin resistance in these rats. In this study, the fatty acid oxidation rate during the vigorous exercise was decreased in prediabetic rats, which was consistent with the findings from a previous clinical study (Cancino Ramírez et al. 2018). This result suggested that prediabetes contributes to impaired fatty acid utilization during exercise, indicating a disruption of metabolic flexibility in an insulin resistant condition (Blaak and Saris 2002; Galgani et al. 2008). Consistent with prior studies, an increase in carbohydrate and fatty acid oxidation rates were indicated in our prediabetic rats on an exercise program (Blaak and Saris 2002; Consitt et al. 2019; Evans et al. 2019; Horowitz et al. 2000). In contrast, caloric restriction failed to increase carbohydrate oxidation rate, and further decreased fatty acid oxidation rate in our prediabetic rats. These findings could be due to a negative energy balance from baseline to follow-up, resulting in a decrease in the oxidization of substrate. Since an increase in aerobic metabolism leads to an increase in oxidative stress which consequently contributes to aging (Liguori et al. 2018), caloric restriction induced decreased substrate oxidation is likely to be another potential mechanism involved in the mediation of caloric restriction-induced increased longevity of life. Our results also showed that not only long-term caloric restriction and exercise, but short-term caloric restriction followed by weight maintenance and short-term exercise followed by weight maintenance also significantly improved cardiac function in prediabetic rats. These findings highlighted that these short-term interventions followed by weight maintenance also exert beneficial effects on the amelioration of cardiac dysfunction in the prediabetic condition.

Consistent with prior studies (Amput et al. 2020a, b; Maneechote et al. 2019; Palee et al. 2019), in our groups prediabetes also resulted in an increase in mitochondrial ROS production, mitochondrial membrane depolarization, and mitochondrial swelling in the heart. Interestingly, caloric restriction exerted greater efficacy in the reduction of mitochondrial ROS production and mitochondrial depolarization than exercise. Complexes I-V protein expressions were lower in prediabetic rats than those of ND rats. These results suggested that increased ROS could contribute to decreased oxidative phosphorylation complex content (Guo et al. 2013). These could be improved by either caloric restriction or exercise. Since it was previously reported that complex I was strongly associated with mitochondrial content (Larsen et al. 2012), a restoration of complex I protein expression in our prediabetic rats receiving all kinds of intervention suggested that both short- and long-term caloric restriction or exercise improved mitochondrial content in prediabetic rats. Unlike other interventions, the long-term caloric restriction failed to restore complexes II and III protein expressions in prediabetic rats. Our results were consistent with a previous study, in which complex III protein expression was not increased after long-term caloric restriction (Olgun et al. 2002). However, the mechanisms mediating these findings remain unclear. Since low protein intake could lead to decreased tissue protein synthesis (Wykes et al. 1996), it is possible that long-term caloric restriction resulted in decreased protein intake, and this might contribute to an impairment of complexes II and III protein restoration. Unlike complexes I-V protein expressions, we found that the heart of prediabetic rats had higher levels of respiratory control ratio than that of ND rats, which was also observed previously in the adipocyte (Böhm et al. 2020) and the heart (Maneechote et al. 2019). This could be due to the HFD-induced nutrient overload in cardiac mitochondria, which consequently led to increased oxygen consumption by the remaining oxidative phosphorylation content in the heart. Prediabetic rats in the long-term exercise group also displayed a higher respiratory control ratio than that of ND rats. This is likely to be due to either prolonged HFD consumption in this group of rats or the direct effect of exercise on increased cardiac oxygen consumption (D. L. Evans 1985). Consistent with the whole-body fatty oxidation rate and the results from previous studies (Ferguson et al. 2007; Serna et al. 2020), a negative energy balance led to a reduction in respiratory control ratio in our prediabetic rats in the caloric restriction groups. This could possibly be due to a greater reduction of mitochondrial ROS production and mitochondrial membrane depolarization in these groups of rats, when compared with those in the exercise groups.

Indicating an improvement in peripheral insulin sensitivity, we showed that either caloric restriction or exercise improved cardiac insulin signaling in prediabetic rats. On the other hand, we found that only long-term interventions could increase p-AMPK/total AMPK protein expression, decrease cardiac apoptosis, decrease cardiac inflammation, and increase antioxidative capacity in the heart back to normal. However, prior studies demonstrated that short-term lifestyle modification could upregulate AMPK phosphorylation, alleviate apoptosis, attenuate inflammation, and improve antioxidative capacity in the heart (Kobara et al. 2015; Ma et al. 2020; Marzetti et al. 2009; Palee et al. 2019; Pinckard et al. 2019; Powers et al. 2014; Shinmura et al. 2005). These contradictory results could be due to the different timepoints of cardiac investigation and/or different experimental models and procedures. For example, we evaluated all cardiac parameters at the weight maintenance phase, which was 10 weeks after the termination of short-term caloric restriction or exercise. In contrast, the previous studies identified immediate effects of short-term lifestyle modification on cardiac health. All of these findings suggested that the therapeutic effects of caloric restriction and exercise on these cardiac parameters are not permanent, as these benefits are not displayed during the weight maintenance period. All of these findings suggested that the therapeutic effects of caloric restriction and exercise on these cardiac parameters are not permanent, as these benefits are not displayed during the weight maintenance period.

Focusing on cardiac inflammation, it has been shown that the inflammatory process is strongly associated with the development of coronary artery disease (Arnold et al. 2021; Golia et al. 2014) and heart failure (Sorriento and Iaccarino 2019). Hence, an attenuation of cardiac inflammation is considered as a key mechanism to either prevent or ameliorate cardiovascular disease. Despite the improvement of blood metabolic parameters and cardiac mitochondrial function, our results showed that neither short-term caloric restriction nor exercise followed by weight maintenance exerted an anti-inflammatory effect on the heart. In fact, these findings implied that short-term behavioral modification followed by weight maintenance alone may insufficiently help to decrease the risk of prediabetes-induced cardiovascular disorders.

Surprisingly, we found that caloric restriction led to a reduction of cardiac ROS level without an attenuation of cardiac lipid peroxidation in our prediabetic rats. In fact, it has been proved that increased ROS level is a major cause of increased lipid peroxidation (Su et al. 2019). Unfortunately, ROS-induced lipid peroxidation can be irreversible (Juan et al. 2021). For this reason, it is highly possible that increased ROS level leads to increased lipid peroxidation in prediabetic rats after 12 weeks of HFD consumption, as were also observed in previous studies (Amput, et al. 2020a, b; Tanajak et al. 2017). However, caloric restriction fails to reverse prediabetes-induced lipid peroxidation despite a reduction of ROS level. Unlike caloric restriction, we observed that exercise could reverse prediabetes-induced cardiac lipid peroxidation, as indicated by a reduction of cardiac MDA level. This effect of exercise may be mediated by an activation of aldehyde dehydrogenase (ALDH) enzyme that plays an important role in the catabolism of MDA (Ayala et al. 2014; Marchitti et al. 2008). To verify this hypothesis, a further study measuring the level of cardiac ALDH enzyme in prediabetic rats after exercise is required.

Our results also demonstrated that there was a significantly positive correlation between CRF level at baseline and at follow-up. These findings support the fact that CRF is highly intrinsic (Schutte et al. 2016). In agreement with previous clinical studies (Brien et al. 2007; Carnethon et al. 2003), our results demonstrated that high CRF could provide protection against metabolic syndrome via the attenuation of obesity, insulin resistance, and dyslipidemia. This was indicated by significant correlations between CRF level at baseline versus anthropometry and metabolic parameters at follow-up in our rats. Interestingly, this protective effect of high CRF was also exhibited in our HFD-fed rats, who had already developed metabolic syndrome as indicated by obesity, prediabetes, and dyslipidemia. High CRF slowed the progression of severe metabolic syndrome following chronic HFD consumption in those rats. Importantly, this benefit of high CRF remained in our prediabetic rats in the short-term caloric restriction followed by weight maintenance, long-term caloric restriction, or short-term exercise followed by weight maintenance groups. These findings suggested that a high level of CRF provides an additional benefit regarding the amelioration of metabolic syndrome in subjects who either have a restriction in calorie intake or participate in short-term exercise. Nonetheless, baseline CRF did not show a correlation with any metabolic parameters at follow-up in the prediabetic long-term exercise group. This finding indicated that chronic exercise overwhelms the benefit of high CRF as regards protection against metabolic syndrome. Therefore, it is highly suggestive that long-term exercise is the most helpful lifestyle modification for prediabetic individuals who have low CRF level.

Regarding the cardioprotective effect of high CRF, our results demonstrated that this benefit is mediated through the modulation of cardiac mitochondrial function, insulin signaling, metabolism, mitochondrial biogenesis, mitochondrial respiration, oxidative phosphorylation, mitochondrial dynamics, apoptosis, antioxidative capacity, inflammation, and lipid peroxidation. Interestingly the cardioprotective effect of high CRF remained in our prediabetic rats, both with and without lifestyle modification. Indeed, a high CRF confers an additional benefit regarding cardioprotection in prediabetes, and this is independent of lifestyle interventions. Hence, an increment of CRF level along with the lifestyle modification is strongly recommended in all prediabetic subjects, in order to achieve the highest level of cardioprotection.

## Conclusion

Under prediabetic conditions, either short-term caloric restriction or exercise followed by weight maintenance exerts a measurable therapeutic effect on cardiometabolic health. Interestingly, high CRF is also associated with protection against cardiometabolic impartment in prediabetes, both with and without lifestyle modification. These results indicate that prediabetic subjects who are unable to perform lifestyle modification may achieve cardiometabolic protection by targeting at increased CRF level. Furthermore, high CRF may provide an additional benefit on cardiometabolic protection in prediabetic subjects undergoing either caloric restriction or exercise. Our findings also suggest that in addition to the use of anti-diabetic drugs and lifestyle modification, development of novel genetic and medical paradigms that directly target an enhancement of CRF level should be conducted. This may contribute to more effective treatment of cardiometabolic impairment in prediabetes.

## Supplementary Information

**Additional file 1.** Additional tables and figures.

## Data Availability

The datasets used and/or analyzed during the current study are available from the corresponding author on reasonable request.

## Abbreviations

ADP: Adenosine diphosphate
ALDH: Aldehyde dehydrogenase;
AMPK: Activated protein kinase
CRF: Cardiorespiratory fitness
DAPI: 4, 6-Diamidino-2-phenylindole
DCFH-DA: Dichlorofluorescein diacetate
DRP1: Dynamin-related protein 1
E/A: Early to late ventricular filling velocity
ELISA: Enzyme-linked immunosorbent assay
HDL: High density lipoprotein
HF: High frequency
HFD: High-fat diet
HOMA-IR: Homeostatic model assessment for insulin resistance
HPLC: High-performance liquid chromatography
HRV: Heart rate variability
IRS: Insulin receptor substrate 1
LDL: Low density lipoprotein
LF: Low frequency
LV: Left ventricle
LVEF: Left ventricular ejection fraction
LVFS: Left ventricular fractional shortening
MDA: Malondialdehyde
MFN1: Mitofusin 1
MFN2: Mitofusin 2
NADH: Nicotinamide adenine dinucleotide
ND: Normal diet
OPA1: Optic atrophy 1
OXPHOS: Oxidative phosphorylation
p-AMPK: Phosphorylated-activated protein kinase
PBS: Phosphate-buffered saline
PDM: Prediabetes with no intervention
PDMCR-SM: Prediabetes with short-term caloric restriction followed by weight maintenance
PDMCR-L: Prediabetes with long-term caloric restriction
PDMEX-SM: Prediabetes with short-term exercise followed by weight maintenance
PDMEX-L: Prediabetes with long-term exercise
p-DRP1^ser616^: Phosphorylated-dynamin-related at serine^616^
PGC-1α: Peroxisome proliferator-activated receptor gamma coactivator-1α
p-IRS: Phosphorylated-insulin receptor substrate 1
ROS: Reactive oxygen species
SDS: Sodium dodecyl sulfate
SOD2: Superoxide dismutase 2
TBARS: Thiobarbituric acid reactive substances
TdT: Terminal deoxynucleotidyl transferase
TNF-α: Tumor necrosis factor-α
TUNEL: Terminal deoxynucleotidyl transferase dUTP nick end labeling
VCO_2_: Volume of exhaled carbon dioxide
VO_2_: Volume of oxygen uptake
VO_2_max: Maximal volume of exhaled oxygen

## References

[CR1] Amput P, Palee S, Arunsak B, Pratchayasakul W, Kerdphoo S, Jaiwongkam T (2020). PCSK9 inhibitor effectively attenuates cardiometabolic impairment in obese-insulin resistant rats. Eur J Pharmacol.

[CR2] Amput P, Palee S, Arunsak B, Pratchayasakul W, Thonusin C, Kerdphoo S (2020). PCSK9 inhibitor and atorvastatin reduce cardiac impairment in ovariectomized prediabetic rats via improved mitochondrial function and Ca(2+) regulation. J Cell Mol Med.

[CR3] Anderson PJ, Critchley JA, Chan JC, Cockram CS, Lee ZS, Thomas GN, Tomlinson B (2001). Factor analysis of the metabolic syndrome: obesity vs insulin resistance as the central abnormality. Int J Obes Relat Metab Disord.

[CR4] Arinno A, Apaijai N, Kaewtep P, Pratchayasakul W, Jaiwongkam T, Kerdphoo S (2019). Combined low-dose testosterone and vildagliptin confers cardioprotection in castrated obese rats. J Endocrinol.

[CR5] Arnold N, Lechner K, Waldeyer C, Shapiro MD, Koenig W (2021). Inflammation and Cardiovascular Disease: The Future. Eur Cardiol.

[CR6] Artero EG, Jackson AS, Sui X, Lee DC, O'Connor DP, Lavie CJ (2014). Longitudinal algorithms to estimate cardiorespiratory fitness: associations with nonfatal cardiovascular disease and disease-specific mortality. J Am Coll Cardiol.

[CR7] Ayala A, Muñoz MF, Argüelles S (2014). Lipid peroxidation: production, metabolism, and signaling mechanisms of malondialdehyde and 4-hydroxy-2-nonenal. Oxid Med Cell Longev.

[CR8] Bansal N (2015). Prediabetes diagnosis and treatment: a review. World J Diabetes.

[CR9] Biesiadecki BJ, Brotto MA, Brotto LS, Koch LG, Britton SL, Nosek TM, Jin JP (2020). Rats genetically selected for low and high aerobic capacity exhibit altered soleus muscle myofilament functions. Am J Physiol Cell Physiol.

[CR10] Blaak EE, Saris WH (2002). Substrate oxidation, obesity and exercise training. Best Pract Res Clin Endocrinol Metab.

[CR11] Böhm A, Keuper M, Meile T, Zdichavsky M, Fritsche A, Häring HU (2020). Increased mitochondrial respiration of adipocytes from metabolically unhealthy obese compared to healthy obese individuals. Sci Rep.

[CR12] Brannick B, Dagogo-Jack S (2018). Prediabetes and cardiovascular disease: pathophysiology and interventions for prevention and risk reduction. Endocrinol Metab Clin North Am.

[CR13] Brien SE, Katzmarzyk PT, Craig CL, Gauvin L (2007). Physical activity, cardiorespiratory fitness and body mass index as predictors of substantial weight gain and obesity: the Canadian physical activity longitudinal study. Can J Public Health.

[CR14] Cai X, Zhang Y, Li M, Wu JH, Mai L, Li J (2020). Association between prediabetes and risk of all cause mortality and cardiovascular disease: updated meta-analysis. BMJ.

[CR15] Cancino Ramírez J, Soto Sánchez J, Zbinden Foncea H, Moreno González M, Leyton Dinamarca B, González Rojas L (2018). Cardiorespiratory fitness and fat oxidation during exercise as protective factors for insulin resistance in sedentary women with overweight or obesity. Nutr Hosp.

[CR16] Carnethon MR, Gidding SS, Nehgme R, Sidney S, Jacobs DR, Liu K (2003). Cardiorespiratory fitness in young adulthood and the development of cardiovascular disease risk factors. JAMA.

[CR17] Chakrabarti P, Kim JY, Singh M, Shin YK, Kim J, Kumbrink J (2013). Insulin inhibits lipolysis in adipocytes via the evolutionarily conserved mTORC1-Egr1-ATGL-mediated pathway. Mol Cell Biol.

[CR18] Chattipakorn N, Incharoen T, Kanlop N, Chattipakorn S (2007). Heart rate variability in myocardial infarction and heart failure. Int J Cardiol.

[CR19] Collins S (1995). The limit of human adaptation to starvation. Nat Med.

[CR20] Consitt LA, Dudley C, Saxena G (2019). Impact of endurance and resistance training on skeletal muscle glucose metabolism in older adults. Nutrients.

[CR21] Domon A, Katayama K, Tochigi Y, Suzuki H (2019). Characterization of novel nonobese type 2 diabetes rat model with enlarged kidneys. J Diabetes Res.

[CR22] Evans DL (1985). Cardiovascular adaptations to exercise and training. Vet Clin North Am Equine Pract.

[CR23] Evans PL, McMillin SL, Weyrauch LA, Witczak CA (2019). Regulation of skeletal muscle glucose transport and glucose metabolism by exercise training. Nutrients.

[CR24] Farinatti P, Castinheiras Neto AG, Amorim PR (2016). Oxygen consumption and substrate utilization during and after resistance exercises performed with different muscle mass. Int J Exerc Sci.

[CR25] Ferguson M, Sohal BH, Forster MJ, Sohal RS (2007). Effect of long-term caloric restriction on oxygen consumption and body temperature in two different strains of mice. Mech Ageing Dev.

[CR26] Forouhi NG, Luan J, Hennings S, Wareham NJ (2007). Incidence of Type 2 diabetes in England and its association with baseline impaired fasting glucose: the Ely study 1990–2000. Diabet Med.

[CR27] Friedewald WT, Levy RI, Fredrickson DS (1972). Estimation of the concentration of low-density lipoprotein cholesterol in plasma, without use of the preparative ultracentrifuge. Clin Chem.

[CR28] Galgani JE, Moro C, Ravussin E (2008). Metabolic flexibility and insulin resistance. Am J Physiol Endocrinol Metab.

[CR29] Ganse B, Ganse U, Dahl J, Degens H (2018). Linear decrease in athletic performance during the human life span. Front Physiol.

[CR30] Gaster M, Beck-Nielsen H (2004). The reduced insulin-mediated glucose oxidation in skeletal muscle from type 2 diabetic subjects may be of genetic origin–evidence from cultured myotubes. Biochim Biophys Acta.

[CR31] Golia E, Limongelli G, Natale F, Fimiani F, Maddaloni V, Pariggiano I (2014). Inflammation and cardiovascular disease: from pathogenesis to therapeutic target. Curr Atheroscler Rep.

[CR32] Gonzalez JT, Green BP, Campbell MD, Rumbold PL, Stevenson EJ (2014). The influence of calcium supplementation on substrate metabolism during exercise in humans: a randomized controlled trial. Eur J Clin Nutr.

[CR33] Gormley SE, Swain DP, High R, Spina RJ, Dowling EA, Kotipalli US, Gandrakota R (2008). Effect of intensity of aerobic training on VO2max. Med Sci Sports Exerc.

[CR34] Guo C, Sun L, Chen X, Zhang D (2013). Oxidative stress, mitochondrial damage and neurodegenerative diseases. Neural Regen Res.

[CR35] Hickner RC, Racette SB, Binder EF, Fisher JS, Kohrt WM (1999). Suppression of whole body and regional lipolysis by insulin: effects of obesity and exercise. J Clin Endocrinol Metab.

[CR36] Hill JO (2006). Understanding and addressing the epidemic of obesity: an energy balance perspective. Endocr Rev.

[CR37] Horowitz JF, Leone TC, Feng W, Kelly DP, Klein S (2000). Effect of endurance training on lipid metabolism in women: a potential role for PPARalpha in the metabolic response to training. Am J Physiol Endocrinol Metab.

[CR38] Hussain SO, Barbato JC, Koch LG, Metting PJ, Britton SL (2001). Cardiac function in rats selectively bred for low- and high-capacity running. Am J Physiol Regul Integr Comp Physiol.

[CR39] Juan CA, Pérez de la Lastra JM, Plou FJ, Pérez-Lebeña E (2021). The Chemistry of Reactive Oxygen Species (ROS) Revisited: Outlining Their Role in Biological Macromolecules (DNA, Lipids and Proteins) and Induced Pathologies. Int J Mol Sci.

[CR40] Karwi QG, Wagg CS, Altamimi TR, Uddin GM, Ho KL, Darwesh AM (2020). Insulin directly stimulates mitochondrial glucose oxidation in the heart. Cardiovasc Diabetol.

[CR41] Khaodhiar L, Cummings S, Apovian CM (2009). Treating diabetes and prediabetes by focusing on obesity management. Curr Diab Rep.

[CR42] Kobara M, Furumori-Yukiya A, Kitamura M, Matsumura M, Ohigashi M, Toba H, Nakata T (2015). Short-term caloric restriction suppresses cardiac oxidative stress and hypertrophy caused by chronic pressure overload. J Card Fail.

[CR43] Koch LG, Kemi OJ, Qi N, Leng SX, Bijma P, Gilligan LJ (2011). Intrinsic aerobic capacity sets a divide for aging and longevity. Circ Res.

[CR44] Kodama S, Saito K, Tanaka S, Maki M, Yachi Y, Asumi M (2009). Cardiorespiratory fitness as a quantitative predictor of all-cause mortality and cardiovascular events in healthy men and women: a meta-analysis. JAMA.

[CR45] Larsen S, Nielsen J, Hansen CN, Nielsen LB, Wibrand F, Stride N (2012). Biomarkers of mitochondrial content in skeletal muscle of healthy young human subjects. J Physiol.

[CR46] Lee DC, Artero EG, Sui X, Blair SN (2010). Mortality trends in the general population: the importance of cardiorespiratory fitness. J Psychopharmacol.

[CR47] Lee J, Cho JY, Kim WK (2014). Anti-inflammation effect of Exercise and Korean red ginseng in aging model rats with diet-induced atherosclerosis. Nutr Res Pract.

[CR48] Liguori I, Russo G, Curcio F, Bulli G, Aran L, Della-Morte D (2018). Oxidative stress, aging, and diseases. Clin Interv Aging.

[CR49] Liu XC, He GD, Lo K, Huang YQ, Feng YQ (2020). The Triglyceride-Glucose Index, an Insulin Resistance Marker, Was Non-linear Associated With All-Cause and Cardiovascular Mortality in the General Population. Front Cardiovasc Med.

[CR50] Ma Z, Qi J, Gao L, Zhang J (2020). Role of Exercise on Alleviating Pressure Overload-Induced Left Ventricular Dysfunction and Remodeling via AMPK-Dependent Autophagy Activation. Int Heart J.

[CR51] Mahat RK, Singh N, Arora M, Rathore V (2019). Health risks and interventions in prediabetes: A review. Diabetes Metab Syndr.

[CR52] Maneechote C, Palee S, Apaijai N, Kerdphoo S, Jaiwongkam T, Chattipakorn SC, Chattipakorn N (2019). Mitochondrial dynamic modulation exerts cardiometabolic protection in obese insulin-resistant rats. Clin Sci (lond).

[CR53] Marchitti SA, Brocker C, Stagos D, Vasiliou V (2008). Non-P450 aldehyde oxidizing enzymes: the aldehyde dehydrogenase superfamily. Expert Opin Drug Metab Toxicol.

[CR54] Marzetti E, Wohlgemuth SE, Anton SD, Bernabei R, Carter CS, Leeuwenburgh C (2009). Cellular mechanisms of cardioprotection by calorie restriction: state of the science and future perspectives. Clin Geriatr Med.

[CR55] Olgun A, Akman S, Serdar MA, Kutluay T (2002). Oxidative phosphorylation enzyme complexes in caloric restriction. Exp Gerontol.

[CR56] Overmyer KA, Evans CR, Qi NR, Minogue CE, Carson JJ, Chermside-Scabbo CJ (2015). Maximal oxidative capacity during exercise is associated with skeletal muscle fuel selection and dynamic changes in mitochondrial protein acetylation. Cell Metab.

[CR57] Palee S, Weerateerangkul P, Chinda K, Chattipakorn SC, Chattipakorn N (2013). Mechanisms responsible for beneficial and adverse effects of rosiglitazone in a rat model of acute cardiac ischaemia-reperfusion. Exp Physiol.

[CR58] Palee S, Minta W, Mantor D, Sutham W, Jaiwongkam T, Kerdphoo S (2019). Combination of exercise and calorie restriction exerts greater efficacy on cardioprotection than monotherapy in obese-insulin resistant rats through the improvement of cardiac calcium regulation. Metabolism.

[CR59] Pifferi F, Aujard F (2019). Caloric restriction, longevity and aging: Recent contributions from human and non-human primate studies. Prog Neuropsychopharmacol Biol Psychiatry.

[CR60] Pinckard K, Baskin KK, Stanford KI (2019). Effects of Exercise to Improve Cardiovascular Health. Front Cardiovasc Med.

[CR61] Pintana H, Apaijai N, Chattipakorn N, Chattipakorn SC (2013). DPP-4 inhibitors improve cognition and brain mitochondrial function of insulin-resistant rats. J Endocrinol.

[CR62] Powers SK, Sollanek KJ, Wiggs MP, Demirel HA, Smuder AJ (2014). Exercise-induced improvements in myocardial antioxidant capacity: the antioxidant players and cardioprotection. Free Radic Res.

[CR63] Pratchayasakul W, Kerdphoo S, Petsophonsakul P, Pongchaidecha A, Chattipakorn N, Chattipakorn SC (2011). Effects of high-fat diet on insulin receptor function in rat hippocampus and the level of neuronal corticosterone. Life Sci.

[CR64] Qureshi WT, Alirhayim Z, Blaha MJ, Juraschek SP, Keteyian SJ, Brawner CA, Al-Mallah MH (2015). Response to letter regarding article, "cardiorespiratory fitness and risk of incident atrial fibrillation: results from the henry ford exercise testing (FIT) Project". Circulation.

[CR65] Ritchie RH, Leo CH, Qin C, Stephenson EJ, Bowden MA, Buxton KD (2013). Low intrinsic exercise capacity in rats predisposes to age-dependent cardiac remodeling independent of macrovascular function. Am J Physiol Heart Circ Physiol.

[CR66] Rochlani Y, Pothineni NV, Kovelamudi S, Mehta JL (2017). Metabolic syndrome: pathophysiology, management, and modulation by natural compounds. Ther Adv Cardiovasc Dis.

[CR67] Sarwer DB, von Sydow Green A, Vetter ML, Wadden TA (2009). Behavior therapy for obesity: where are we now?. Curr Opin Endocrinol Diabetes Obes.

[CR68] Schutte NM, Nederend I, Hudziak JJ, Bartels M, de Geus EJ (2016). Twin-sibling study and meta-analysis on the heritability of maximal oxygen consumption. Physiol Genomics.

[CR69] Serna JDC, Caldeira da Silva CC, Kowaltowski AJ (2020). Functional changes induced by caloric restriction in cardiac and skeletal muscle mitochondria. J Bioenerg Biomembr.

[CR70] Shinmura K, Tamaki K, Bolli R (2005). Short-term caloric restriction improves ischemic tolerance independent of opening of ATP-sensitive K+ channels in both young and aged hearts. J Mol Cell Cardiol.

[CR71] Sorriento D, Iaccarino G (2019). Inflammation and Cardiovascular Diseases: The Most Recent Findings. Int J Mol Sci.

[CR72] Souza RWA, Alves CRR, Medeiros A, Rolim N, Silva GJJ, Moreira JBN (2018). Differential regulation of cysteine oxidative post-translational modifications in high and low aerobic capacity. Sci Rep.

[CR73] Su LJ, Zhang JH, Gomez H, Murugan R, Hong X, Xu D (2019). Reactive Oxygen Species-Induced Lipid Peroxidation in Apoptosis, Autophagy, and Ferroptosis. Oxid Med Cell Longev.

[CR74] Tanajak P, Pintana H, Siri-Angkul N, Khamseekaew J, Apaijai N, Chattipakorn SC, Chattipakorn N (2017). Vildagliptin and caloric restriction for cardioprotection in pre-diabetic rats. J Endocrinol.

[CR75] Thonusin C, Apaijai N, Jaiwongkam T, Kerdphoo S, Arunsak B, Amput P (2019). The comparative effects of high dose atorvastatin and proprotein convertase subtilisin/kexin type 9 inhibitor on the mitochondria of oxidative muscle fibers in obese-insulin resistant female rats. Toxicol Appl Pharmacol.

[CR76] Wang R, Tian H, Guo D, Tian Q, Yao T, Kong X (2020). Impacts of exercise intervention on various diseases in rats. J Sport Health Sci.

[CR77] Wykes LJ, Fiorotto M, Burrin DG, Del Rosario M, Frazer ME, Pond WG, Jahoor F (1996). Chronic low protein intake reduces tissue protein synthesis in a pig model of protein malnutrition. J Nutr.

[CR78] Yokoyama H, Emoto M, Fujiwara S, Motoyama K, Morioka T, Komatsu M (2004). Quantitative insulin sensitivity check index and the reciprocal index of homeostasis model assessment are useful indexes of insulin resistance in type 2 diabetic patients with wide range of fasting plasma glucose. J Clin Endocrinol Metab.

