# Supplementary material for: Effectiveness of high cardiorespiratory fitness in cardiometabolic protection in prediabetic rats
Source: Mol Med. 2022 Mar 10;28:31. doi: 10.1186/s10020-022-00458-9 (PMC8908596; doi:10.1186/s10020-022-00458-9)

**Additional file 1: Table S1 Primary and secondary antibodies for protein expression analysis**

| **Antibodies** | **Sources** | **Identifiers** |
| --- | --- | --- |
| **Primary antibodies for protein expression analysis** | | |
| Anti-AKT | Cell Signaling Technology | Cat: 9272 |
| Anti-p-AKT | Cell Signaling Technology | Cat: 9271 |
| Anti-AMPK | Cell Signaling Technology | Cat: 2532 |
| Anti-p-AMPK | EMD millipore | Cat: 07-681 |
| Anti-cytochrome c | Cell Signaling Technology | Cat: 4272 |
| Anti-DRP1 | Cell Signaling Technology | Cat: 5391 |
| Anti-p-DRP1^Ser616^ | Cell Signaling Technology | Cat: 3455 |
| Anti-GAPDH | Abcam | Cat: ab181602 |
| Anti-IRS | Cell Signaling Technology | Cat: 2382 |
| Anti-p-IRS | Cell Signaling Technology | Cat: 2381 |
| Anti-MFN1 | Abcam | Cat: ab104274 |
| Anti-MFN2 | Cell Signaling Technology | Cat: ab9482 |
| Anti-OPA1 | Cell Signaling Technology | Cat: 80471 |
| Anti-OXPHOS cocktail for rodent | Abcam | Cat: ab110413 |
| Anti-PGC-1α | Abcam | Cat: ab54481 |
| Anti-SOD2 | Cell Signaling Technology | Cat: 13194 |
| Anti-TNF-α | Abcam | Cat: ab9635 |
| Anti-VDAC | Cell Signaling Technology | Cat: 4661 |
| **Secondary antibodies for protein expression analysis** | | |
| Anti- mouse IgG, HRP-linked antibody | Cell Signaling Technology | Cat: 7076S |
| Anti-rabbit IgG, HRP-linked antibody | Cell Signaling Technology | Cat: 7074 |

**Additional file 1: Fig. S1 Western blot for OXPHOS protein expression**

**
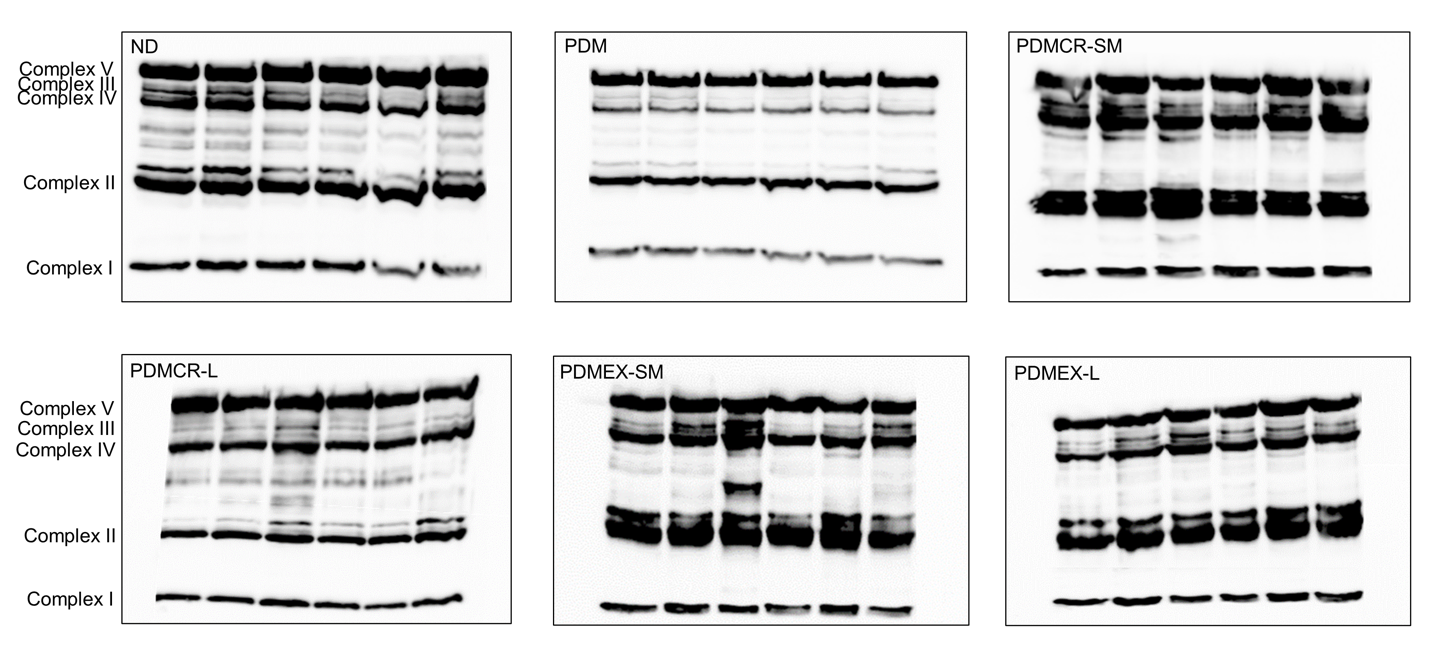
**

**Additional file 1: Fig. S2 Western blot for p-AKT, total AKT, p-IRS, total IRS, p-AMPK, total AMPK and PGC-1α protein expressions**

**
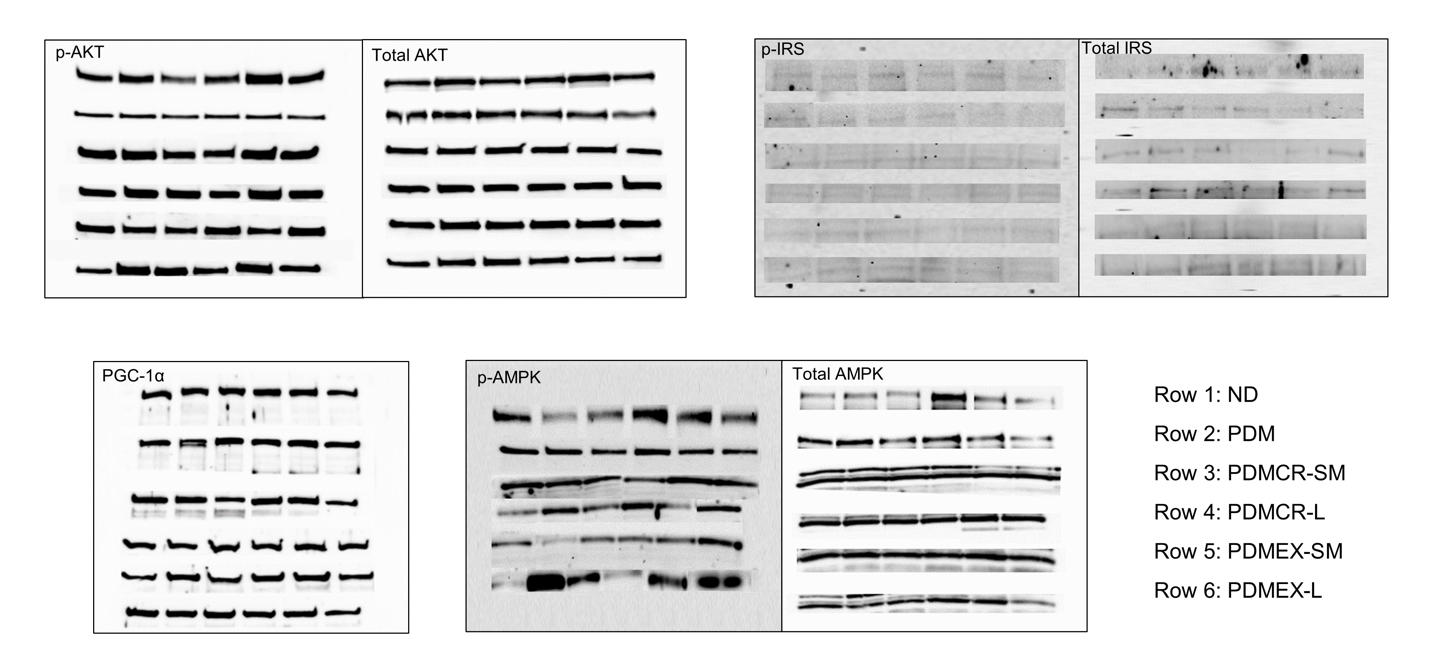
**

**Additional file 1: Fig. S3 Western blot for MFN1,MFN2, OPA1, mitochondrial DRP1, cytosolic p-DRP1^ser616^, and cytosolic total DRP1 protein expressions**

**
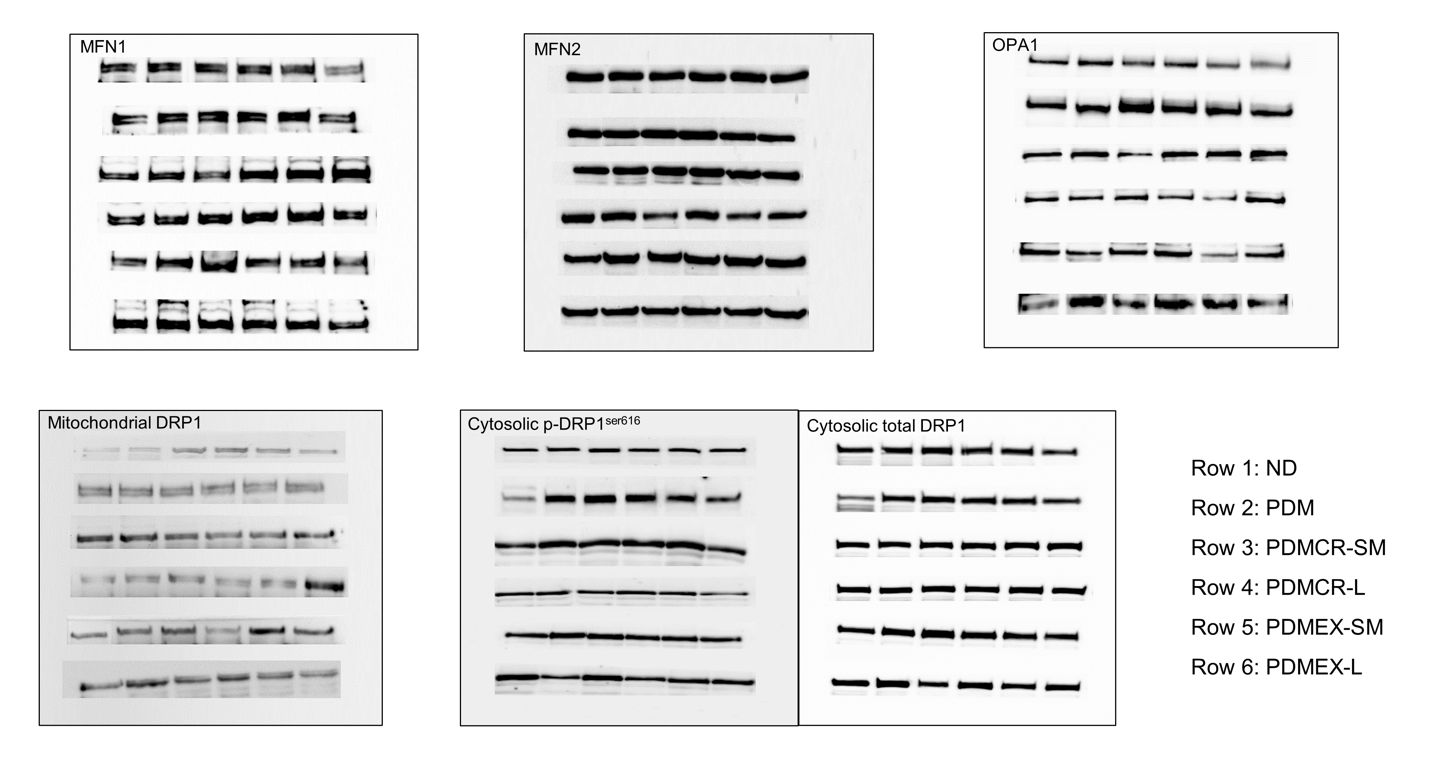
**

**Additional file 1: Fig. S4 Western blot for cytosolic cytochrome c, mitochondrial cytochrome c, TNF-α, SOD2, VDAC, and GAPDH protein expressions**


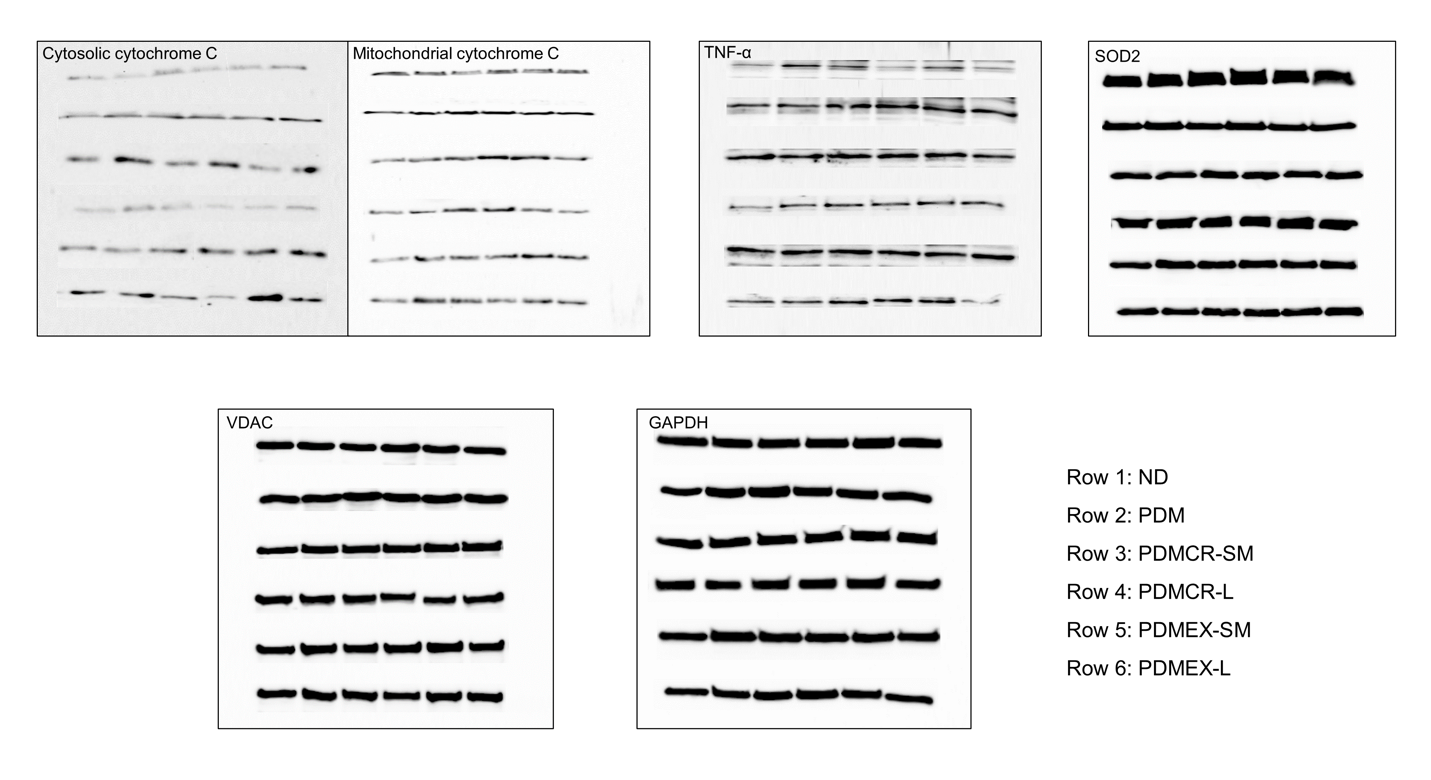

Supplement: Supplementary file 1 — Additional file 1. Additional tables and figures. [file 10020_2022_458_MOESM1_ESM.docx]
